# The changing face of nicotine use in England: Age‐specific annual trends, 2014 to 2024

**DOI:** 10.1111/add.70243

**Published:** 2025-12-07

**Authors:** Sarah E. Jackson, Lion Shahab, Vera Buss, Harry Tattan‐Birch, Sharon Cox, Eve Taylor, Jamie Brown

**Affiliations:** ^1^ Department of Behavioural Science and Health University College London London UK; ^2^ Behavioural Research UK London UK

**Keywords:** cigarettes, e‐cigarettes, heated tobacco, nicotine pouches, smoking, tobacco, vaping

## Abstract

**Aims:**

To examine age‐specific trends in patterns of nicotine use in England between 2014 and 2024, including types of products used, exclusive and dual use of smoking and vaping, smoking frequency and the smoking history of those who vape.

**Design:**

Repeat monthly cross‐sectional analysis of data from a nationally representative survey (the Smoking Toolkit Study).

**Setting:**

England, 2014–2024.

**Participants:**

217 433 adults (≥18y).

**Measurements:**

Prevalence of (non‐medicinal) nicotine use overall and by product type (combustible tobacco, e‐cigarettes, heated tobacco products and nicotine pouches), exclusive and dual use of smoking and vaping, daily versus non‐daily smoking and smoking history among those who vape. Estimates were stratified by age group (18–24, 25–34, 35–44, 45–54, 55–64, ≥65y) and year. Prevalence ratios (PR) with 95% confidence intervals (CI) were calculated to quantify relative changes in prevalence from 2014 to 2024.

**Findings:**

Nicotine use patterns varied markedly by age. Among 18–24‐year‐olds, vaping prevalence increased fivefold, from 5.0% in 2014 to 25.0% in 2024 (PR = 5.00; 95% CI = 4.18–5.91), surpassing smoking by 2023. This contributed to an overall increase in nicotine use (26.1% to 36.5%; PR = 1.40; 95% CI = 1.29–1.53), despite declining smoking rates (25.3% to 19.9%; PR = 0.79; 95% CI = 0.71–0.88). In this age group, exclusive vaping became the most common mode of nicotine use, while nicotine pouch use also increased. Daily smoking declined substantially among 18–24‐year‐olds who smoked, with a shift toward non‐daily smoking. Similar trends were observed among adults aged 25–44, though changes were smaller with increasing age. In older age groups (≥45), daily smoking declined modestly while vaping rose gradually, but there was little overall change in the prevalence of nicotine use. Most adults who vaped had a history of smoking, but the proportion who had never regularly smoked increased, particularly among 18–24‐year‐olds (4.3% to 34.3%; PR = 7.98; 95% CI = 4.56–26.2).

**Conclusions:**

Generational shifts in nicotine use are occurring in England. Nicotine use has risen among young adults over the past decade, but they are increasingly moving away from daily cigarette smoking towards vaping or non‐daily smoking. While older adults have also shown movement away from daily smoking, traditional smoking patterns remain more prevalent in this group. These trends suggest vaping may gradually replace smoking as the dominant form of nicotine consumption.

## INTRODUCTION

The landscape of nicotine use in England has changed substantially in recent years. For decades, nicotine consumption occurred almost exclusively through tobacco smoking (primarily cigarettes) and smoking prevalence had been declining steadily [1]. The emergence and rapid uptake of electronic cigarettes (e‐cigarettes or vapes) [2] has since introduced a new dimension to population‐level patterns of nicotine consumption. E‐cigarettes offer a less harmful alternative to combustible tobacco products [3] and initially, they were primarily used as a harm reduction tool by people who smoked [2]. However, their growing daily use—particularly among young adults and individuals who have never smoked [2, 4, 5, 6]—has sparked debate about their longer‐term public health impact. Debates have centred on the potential for vaping to support smoking cessation, while also potentially contributing to nicotine uptake in those who might not otherwise have used it [7]. Other novel nicotine products have also been introduced to the market in recent years, including modern heated tobacco products (electronic devices that heat processed tobacco with the aim of avoiding combustion) and oral nicotine pouches (tobacco‐free pouches placed in the mouth to release nicotine) [8].

The United Kingdom (UK) government's Tobacco and Vapes Bill [9], which is currently progressing through parliament with cross‐party support [10], aims to create a smokefree generation while addressing growing concerns around youth vaping. The bill proposes landmark measures, including a ban on all tobacco sales to anyone born on or after 1 January 2009 [9]. This progressive age‐of‐sale policy will cover all tobacco‐containing products, but not tobacco‐free nicotine products such as e‐cigarettes and nicotine pouches. The bill also includes powers to implement tighter restrictions on the marketing, packaging and availability of vaping products and other tobacco‐free nicotine products (e.g. pouches), subject to consultation and secondary legislation [9]. As policymakers seek to strike a balance between supporting smoking cessation and preventing uptake of smoking and vaping, timely evidence on age‐specific nicotine use trends is important to inform effective and proportionate regulation.

Understanding how patterns of smoking, vaping and use of other nicotine products are evolving across age groups can provide insights into the future trajectory of nicotine use. Generational differences—shaped by historical exposures, social norms and policy environments—reveal not only how behaviours have evolved, but also where they may be headed [11, 12, 13, 14, 15]. In particular, the smoking and vaping patterns of today's young adults are especially informative, offering a window into the future composition of the nicotine‐using population.

This study, therefore, aimed to describe how nicotine use among adults in England has evolved over the past decade. Using nationally representative data from the monthly cross‐sectional Smoking Toolkit Study, we examined age‐specific annual trends in the prevalence and patterns of nicotine use between 2014 and 2024, including the types of products used and distinctions between exclusive and dual use of smoking and vaping, daily and non‐daily smoking and the smoking history of adults who vape.

## METHODS

### Design

The Smoking Toolkit Study is an ongoing monthly cross‐sectional survey designed to monitor trends in smoking in England [16, 17]. Full details of the study's methodology are available elsewhere [2, 16, 17]. Briefly, the study uses a hybrid of random probability and simple quota sampling to select a new sample of approximately 1700 people 16 years and older across England each month. Raking is used to weight the sample to match the population in England [16]. This profile is determined each month by combining data from the UK Census, the Office for National Statistics mid‐year estimates and the annual National Readership Survey [16]. Comparisons with other national surveys and cigarette sales data have shown the survey produces nationally representative estimates of key socio‐demographic and smoking indices [16, 18].

The survey began in November 2006 and has been conducted each month since, except for December 2008 and April 2020. Data were collected face‐to‐face up to the start of the coronavirus disease 2019 pandemic and via telephone from April 2020 onward. After social distancing restrictions lifted, we collected data via face‐to‐face and telephone surveys in concurrent waves, and the two modes generally show good comparability [19, 20]. The lower age limit was raised to 18 years between April 2020 and December 2021.

The present analyses used data collected between January 2014 and December 2024 (the earliest and most recent complete years of data including vaping status available at the time of analysis). We restricted the sample to adults ≥18 years old (the legal age of sale of tobacco and e‐cigarettes) for consistency across the time series and aggregated data annually for analysis.

### Measures

Smoking status was assessed by asking participants which of the following statements best applied to them: (a) I smoke cigarettes (including hand‐rolled) every day; (b) I smoke cigarettes (including hand‐rolled), but not every day; (c) I do not smoke cigarettes at all, but I do smoke tobacco of some kind (e.g. pipe, cigar or shisha); (d) I have stopped smoking completely in the last year; (e) I stopped smoking completely more than a year ago; and (f) I have never been a smoker (i.e. smoked for a year or more). For analysis, responses were categorised as follows:

- Current smoking: (a)–(c)
- Daily cigarette smoking: (a)
- Non‐daily cigarette smoking: (b)
- Exclusive non‐cigarette tobacco smoking: (c)
- Recent former smoking: (d)
- Long‐term former smoking: (e)
- Never regular smoking: (f)

Vaping status was assessed within several questions asking about use of a range of nicotine products. Participants reporting current smoking were asked ‘Do you regularly use any of the following in situations when you are not allowed to smoke?’ and those who reported cutting down ‘Which, if any, of the following are you currently using to help you cut down the amount you smoke?’; those reporting current smoking or recent former smoking were asked ‘Can I check, are you using any of the following either to help you stop smoking, to help you cut down or for any other reason at all?’; and those reporting long‐term former smoking or never regular smoking were asked ‘Can I check, are you using any of the following?’ We defined current vaping as reporting using an e‐cigarette in response to any of these questions.

Use of heated tobacco products and nicotine pouches was assessed within the same questions as vaping status. We defined heated tobacco use as reporting using a ‘heat‐not‐burn cigarette (e.g. iQOS, heatsticks)’ and nicotine pouch use as reporting using ‘tobacco‐free nicotine pouch/pod or white pouches that you place on your gum (e.g. Zyn, On!, Nordic Spirit, Velo, Lyft and Skruf)’ in response to any of these questions. As these products are relatively new, their use was not assessed across the entire period. Analyses of these variables were, therefore, limited to 2017 to 2024 for heated tobacco use and to 2021 to 2024 for pouch use (the only complete years of data to assess use of these products).

Nicotine use was defined as current smoking, vaping, heated tobacco use or nicotine pouch use. We did not include the use of nicotine replacement therapy (NRT) (e.g. nicotine patches, gum, lozenges or inhalators) because these are regulated as medicinal rather than consumer products (available in England on prescription or over‐the‐counter).

Among those who smoked or vaped, exclusive smoking was defined as current smoking and no current vaping; exclusive vaping was defined as current vaping and no current smoking; and dual use was defined as both current smoking and current vaping.

Age was categorised as 18 to 24, 25 to 34, 35 to 44, 45 to 54, 55 to 64 and ≥65 years. Survey year was categorised as 2014 through 2024.

### Statistical analysis

Analyses were conducted using R v.4.4.1. The analyses were not pre‐registered and should be considered exploratory. We excluded participants with missing data on smoking or vaping. Given the very low proportion of missingness (see Results), exclusion of these cases is unlikely to introduce bias and we judged complete‐case analysis appropriate for the study. All analyses were run on weighted data, but sample sizes are reported unweighted to provide transparency about the actual number of participants contributing to the analysis (see Data S1).

Within each age group and year, we estimated: (a) among adults, the overall prevalence of nicotine use and the prevalence of smoking, vaping, heated tobacco use and nicotine pouch use; (b) among adults who smoke or vape, the proportion exclusively smoking, exclusively vaping or dual using; (c) among adults who smoke, the proportion using each type and pattern of smoking (daily cigarette, non‐daily cigarette or exclusive non‐cigarette tobacco); and (d) among adults who vape, the proportion with each smoking status (current, recent former, long‐term former or never regular smoking). We presented results graphically, providing annual estimates with 95% CI within each age group in Data S2–S5. We also reported absolute estimates of the proportion of adults exclusively smoking, exclusively vaping and dual using in Data S3 and the proportion of adults smoking cigarettes daily, smoking cigarettes non‐daily and exclusively smoking non‐cigarette tobacco in Data S4.

For each outcome assessed over the whole period (all except prevalence of heated tobacco and nicotine pouch use), we reported relative and absolute changes from the start to the end of the study period alongside 95% CIs calculated using bootstrapping (500 replications). Relative changes were reported as prevalence ratios (PR) (calculated as prevalence in 2024 divided by prevalence in 2014) (Table 1) and absolute changes as percentage point changes (calculated as prevalence in 2024 minus prevalence in 2014) (Table 2). We followed the ‘New Statistics' approach to reporting and interpretation of results [21, 22], avoiding dichotomous thinking about statistical significance (i.e. whether a change in prevalence is significant or not). Instead, we presented effect sizes with CIs, described differences across age groups and indicated uncertainty where 95% CIs overlapped 1.

**TABLE 1:** Age‐specific relative changes in smoking and vaping patterns among adults in England, 2014 to 2024.

Age group (y)	Relative change from 2014 to 2024, prevalence ratio^a^ [95% CI]^b^					
	18–24	25–34	35–44	45–54	55–64	≥65
Among adults						
Nicotine use^c^	1.40 [1.29–1.53]	1.30 [1.20–1.40]	1.21 [1.09–1.34]	1.01 [0.92–1.12]	1.09 [0.98–1.23]	1.11 [0.97–1.24]
Smoking	0.79 [0.71–0.88]	0.85 [0.77–0.95]	0.86 [0.77–0.96]	0.78 [0.69–0.87]	0.85 [0.75–0.97]	0.90 [0.78–1.03]
Vaping	5.00 [4.18–5.91]	3.09 [2.67–3.66]	2.37 [1.94–2.83]	1.72 [1.44–2.14]	1.88 [1.56–2.46]	1.77 [1.40–2.33]
Among adults who smoke or vape						
Exclusive smoking	0.37 [0.32–0.42]	0.53 [0.48–0.58]	0.64 [0.59–0.70]	0.73 [0.66–0.80]	0.77 [0.70–0.85]	0.83 [0.76–0.90]
Exclusive vaping	14.6 [9.66–25.3]	6.54 [4.94–8.98]	3.83 [2.98–5.29]	4.10 [3.03–5.73]	4.32 [3.04–6.68]	3.95 [2.79–5.82]
Dual use	1.64 [1.34–2.02]	1.25 [1.02–1.55]	1.05 [0.82–1.31]	0.94 [0.76–1.19]	0.82 [0.63–1.11]	0.79 [0.56–1.08]
Among adults who smoke						
Daily cigarette smoking	0.55 [0.50–0.62]	0.73 [0.68–0.79]	0.75 [0.70–0.81]	0.84 [0.79–0.90]	0.89 [0.84–0.94]	0.87 [0.81–0.92]
Non‐daily cigarette smoking	2.99 [2.43–3.74]	2.23 [1.75–2.97]	2.11 [1.64–2.73]	1.89 [1.37–2.81]	1.49 [1.04–2.13]	2.25 [1.40–3.59]
Non‐cigarette tobacco smoking	9.90 [4.77–22.3]	8.33 [4.44–18.5]	5.00 [2.77–12.8]	3.07 [1.78–6.02]	3.08 [1.82–6.37]	1.66 [1.03–2.87]
Among adults who vape						
Never regular smoking	7.98 [4.56–26.2]	8.87 [4.12–2.2 m]^d^	2.13 [0.96–7.35]	2.17 [0.98–7.00]	2.81 [0.77–424 k]^d^	2.21 [0.40–252 k]^4^
Long‐term (≥1 y) former smoking	3.78 [1.82–12.4]	3.32 [2.29–5.53]	4.67 [2.99–8.84]	4.21 [2.76–7.15]	3.45 [2.36–6.04]	3.97 [2.46–8.03]
Recent (<1 y) former smoking	1.37 [0.69–3.83]	1.10 [0.70–2.15]	0.53 [0.34–0.88]	0.59 [0.33–1.21]	0.76 [0.36–1.85]	0.49 [0.17–1.32]
Current smoking	0.45 [0.39–0.52]	0.51 [0.45–0.59]	0.53 [0.43–0.63]	0.55 [0.46–0.65]	0.47 [0.37–0.58]	0.48 [0.38–0.61]

Abbreviation: PR = prevalence ratios.

^a^ PR were calculated as the prevalence in 2024 divided by the prevalence in 2014. Values <1 indicate an overall decrease in prevalence between 2014 and 2024 (e.g. PR = 0.60 indicates a 40% decrease) and values >1 indicate an overall increase (e.g. PR = 1.40 indicates a 40% increase).

^b^ 95% CI were calculated using bootstrapping with 500 replications.

^c^ Defined as smoking, vaping, heated tobacco use or nicotine pouch use. Prevalence ratios are not reported separately for heated tobacco or nicotine pouches because use of these products was not assessed over the whole period.

^d^ 95% CIs were very wide for the 25–34 years, 55–65 years and ≥65 years age groups, because of low baseline prevalence and high relative changes over time (Data S5).

**TABLE 2:** Age‐specific absolute changes in smoking and vaping patterns among adults in England, 2014 to 2024.

Age group (y)	Absolute change from 2014 to 2024, percentage points^a^ [95% CI]^b^					
	18–24	25–34	35–44	45–54	55–64	≥65
Among adults						
Nicotine use^c^	10.4 [7.79– 13.2]	8.09 [5.57– 10.37]	4.72 [2.19– 7.16]	0.31 [−1.76 to 2.51]	1.73 [−0.43 to 3.91]	1.01 [−0.34 to 2.31]
Smoking	−5.35 [−7.70 to −2.91]	−3.83 [−6.19 to −1.30]	−2.85 [−4.93 to −0.82]	−4.52 [−6.52 to −2.58]	−2.54 [−4.47 to −0.46]	−0.94 [−2.27 to 0.28]
Vaping	19.9 [17.76–22.3]	14.0 [12.2–16.0]	8.16 [6.39–9.93]	4.33 [2.90–6.05]	3.85 [2.71–5.28]	1.70 [0.98–2.51]
Among adults who smoke or vape						
Exclusive smoking	−51.1 [−56.0 to −46.0]	−35.8 [−40.2 to −31.4]	−26.2 [−30.8 to −21.0]	−19.9 [−25.1 to −14.1]	−17.7 [−23.2 to −11.2]	−13.4 [−19.3 to −7.31]
Exclusive vaping	40.7 [37.2–44.6]	31.0 [27.4–34.7]	25.3 [21.2–30.0]	21.0 [16.8–25.3]	20.9 [16.5–25.6]	16.8 [12.8–20.9]
Dual use	10.4 [6.27–15.0]	4.77 [0.34–9.40]	0.92 [−3.65 to 4.93]	−1.17 [−5.65 to 3.71]	−3.21 [−7.30 to 1.57]	−3.43 [−7.73 to 1.04]
Among adults who smoke						
Daily cigarette smoking	−37.7 [−43.2 to −32.1]	−23.5 [−27.9 to −18.0]	−21.2 [−26.3 to −16.1]	−13.9 [−19.1 to −8.93]	−9.86 [−14.8 to −5.20]	−11.8 [−17.2 to −6.67]
Non‐daily cigarette smoking	28.8 [22.6–34.8]	14.7 [9.99–19.5]	14.0 [8.90–18.7]	8.28 [3.87–13.0]	4.87 [0.49–9.02]	8.06 [3.31–12.2]
Non‐cigarette tobacco smoking	8.91 [5.82–11.9]	8.82 [5.91–11.7]	7.19 [4.60–10.1]	5.58 [2.72–9.19]	4.99 [2.35–7.74]	3.69 [0.22–7.21]
Among adults who vape						
Never regular smoking	30.0 [24.8–35.6]	11.8 [8.69–14.9]	5.06 [−0.26 to 9.59]	3.43 [−0.13 to 7.41]	2.86 [−0.62 to 6.45]	2.29 [−2.27 to 6.97]
Long‐term (≥1 y) former smoking	14.2 [8.02–19.5]	24.9 [18.9–30.9]	35.2 [27.9–41.9]	35.0 [27.0–42.1]	38.4 [29.4–48.1]	41.3 [30.3–52.5]
Recent (<1 y) former smoking	2.34 [−3.06 to 7.31]	1.18 [−4.46 to 6.94]	−8.93 [−16.0 to −1.57]	−4.57 [−10.0 to 1.34]	−2.19 [−8.80 to 3.89]	−5.46 [−13.0 to 1.61]
Current smoking	−46.5 [−54.2 to −38.6]	−37.9 [−44.8 to −30.4]	−31.3 [−41.5 to −22.7]	−33.9 [−42.4 to −25.7]	−39.1 [−49.9 to −28.3]	−38.2 [−47.9 to −25.7]

^a^ Absolute percentage point changes were calculated as the prevalence in 2024 minus the prevalence in 2014. Values <0 indicate an overall decrease in prevalence between 2014 and 2024 and values >0 indicate an overall increase.

^b^ 95% CI were calculated using bootstrapping with 500 replications.

^c^ Defined as smoking, vaping, heated tobacco use or nicotine pouch use. Changes are not reported separately for heated tobacco or nicotine pouches because use of these products was not assessed over the whole period.

## RESULTS

There were 218 214 adults ≥18 years old in England surveyed between January 2014 and December 2024. We excluded 784 (0.4%) with missing data on smoking or vaping status, leaving a final sample of 217 433 participants [weighted mean (SD) age = 49.6 (19.0) years; 49.8% women]. Sample sizes by age group and year are provided in Data S1.

### Prevalence of nicotine use, smoking, vaping, heated tobacco use and nicotine pouch use

**FIGURE 1:**
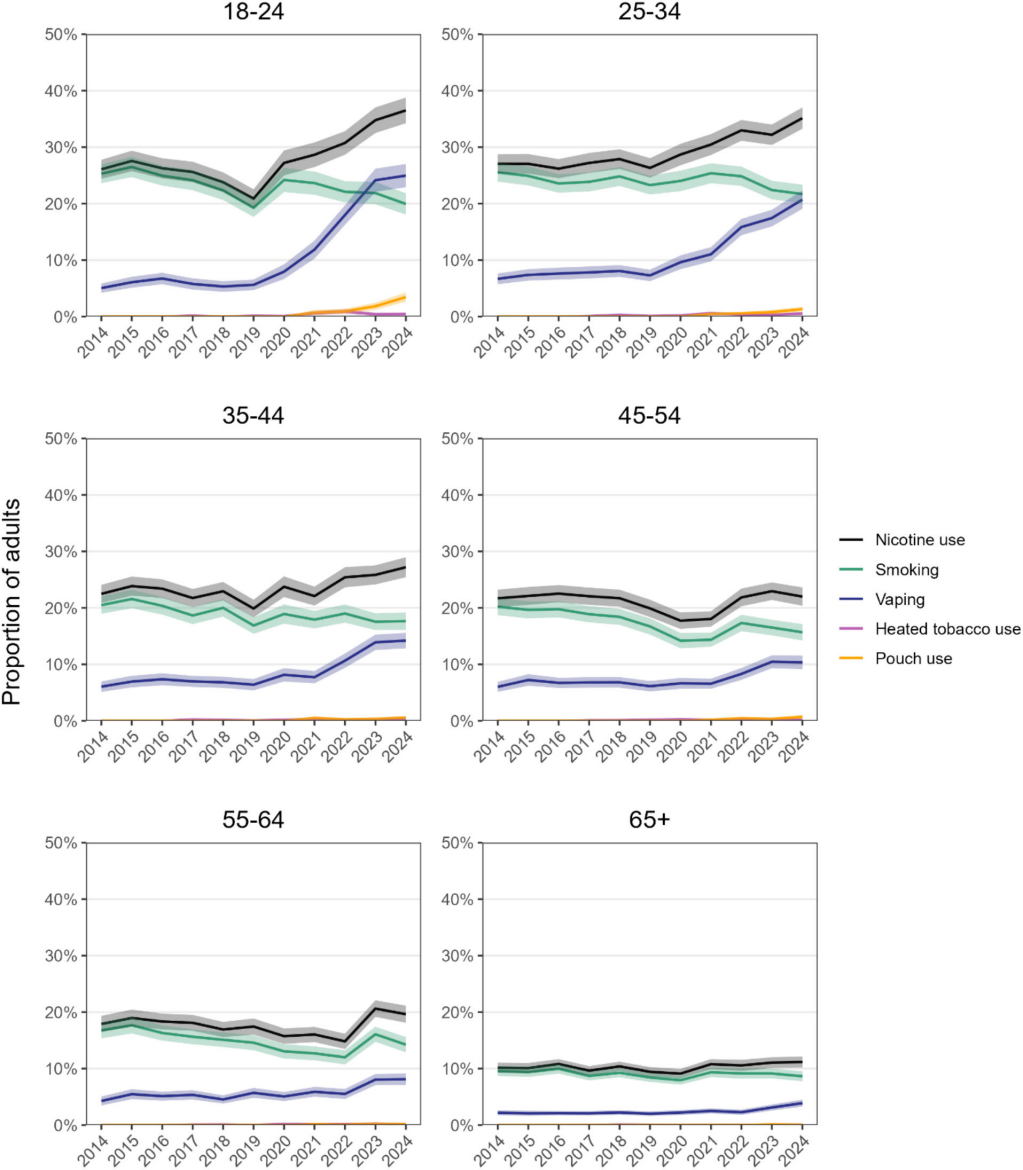
Prevalence of nicotine use, smoking, vaping, heated tobacco use and nicotine pouch use among adults in England, 2014 to 2024. Lines represent weighted prevalence by year. Shaded bands represent 95% CI. Nicotine use is defined as smoking, vaping, heated tobacco use, or nicotine pouch use. Data are provided in tabular form in Data S2.

Between 2014 and 2024, patterns of nicotine use varied considerably by age group (Figure 1, Tables 1, 2, Data S2). Among adults 18 to 24 years old, overall nicotine use declined from 2014 to 2019, primarily because of a steady decrease in smoking, while vaping remained relatively stable. In 2020, smoking prevalence increased, before resuming its downward trend. Meanwhile, the decline in nicotine use reversed as vaping surged in 2021. Vaping prevalence increased fivefold over the decade [from 5.0% in 2014 to 25.0% in 2024; PR = 5.00 (95% CI = 4.18–5.91)], overtaking smoking by 2023 and contributing to an overall rise in nicotine use [from 26.1% to 36.5%; PR = 1.40 (1.29–1.53)] despite a decline in smoking [from 25.3% to 19.9%; PR = 0.79 (0.71–0.88)]. There was also a smaller increase in use of nicotine pouches (from 0.7% in 2021 to 3.4% in 2024), particularly since 2023, while use of heated tobacco products remained rare (<1%).

Similar, although less pronounced, changes were observed among adults 25 to 34 years old. Smoking declined [from 25.5% in 2014 to 21.7% in 2024; PR = 0.85 (0.77–0.95)], while vaping prevalence tripled [from 6.7% to 20.7%; PR = 3.09 (2.67–3.66)], nearly reaching parity with smoking by 2024. The rise in vaping more than offset the decline in smoking, contributing to an overall rise in nicotine use [from 27.0% to 35.1%; PR = 1.30 (1.20–1.40)]. Use of heated tobacco products and nicotine pouches also increased, but remained rare (0.5% and 1.3%, respectively, in 2024).

Among those age 35 to 44 years old, comparable trends were observed, although changes were ever more modest. The overall prevalence of nicotine use rose only slightly [from 22.5% to 27.2%; PR = 1.21 (1.09–1.34)]. Smoking continued to decline [from 20.5% to 17.6%; PR = 0.86 (0.77–0.96)], while vaping more than doubled [from 6.0% to 14.2%; PR = 2.37 (1.94–2.83)], suggesting a slower but ongoing shift in product use. Use of heated tobacco products or nicotine pouches remained low and relatively consistent over time in this age group (0.4% and 0.6%, respectively, in 2024).

Among older age groups (≥45), nicotine use generally remained stable over the decade. More modest increases in vaping were offset by declining smoking rates, with minimal impact from heated tobacco products or nicotine pouches, which remained uncommon throughout. Among those age 45 to 54 years old, smoking declined [from 20.2% to 15.7%; PR = 0.78 (0.69–0.87)], while vaping increased [from 6.0% to 10.3%; PR = 1.72 (1.44–2.14)], but remained less prevalent than in younger groups. Among those age 55 to 64 years old, smoking followed a downward trajectory overall [from 16.8% to 14.2%; PR = 0.85 (0.75–0.97)], although this was disrupted by a slight uptick in the final years of the study period (2022–2024), suggesting a potential reversal or plateau in progress. Meanwhile, vaping prevalence increased slowly [from 4.3% to 8.1%; PR = 1.88 (1.56–2.46)], but remained less common than smoking. Among those age ≥65 years old, both smoking and vaping remained comparatively lower and more stable throughout the study period (≤10.0% and ≤3.9%, respectively), although there was evidence of a gradual increase in vaping [from 2.2% to 3.9%; PR = 1.77 (1.40–2.33)], predominantly in the latter part of the period.

### Patterns of exclusive and dual use of smoking and vaping

**FIGURE 2:**
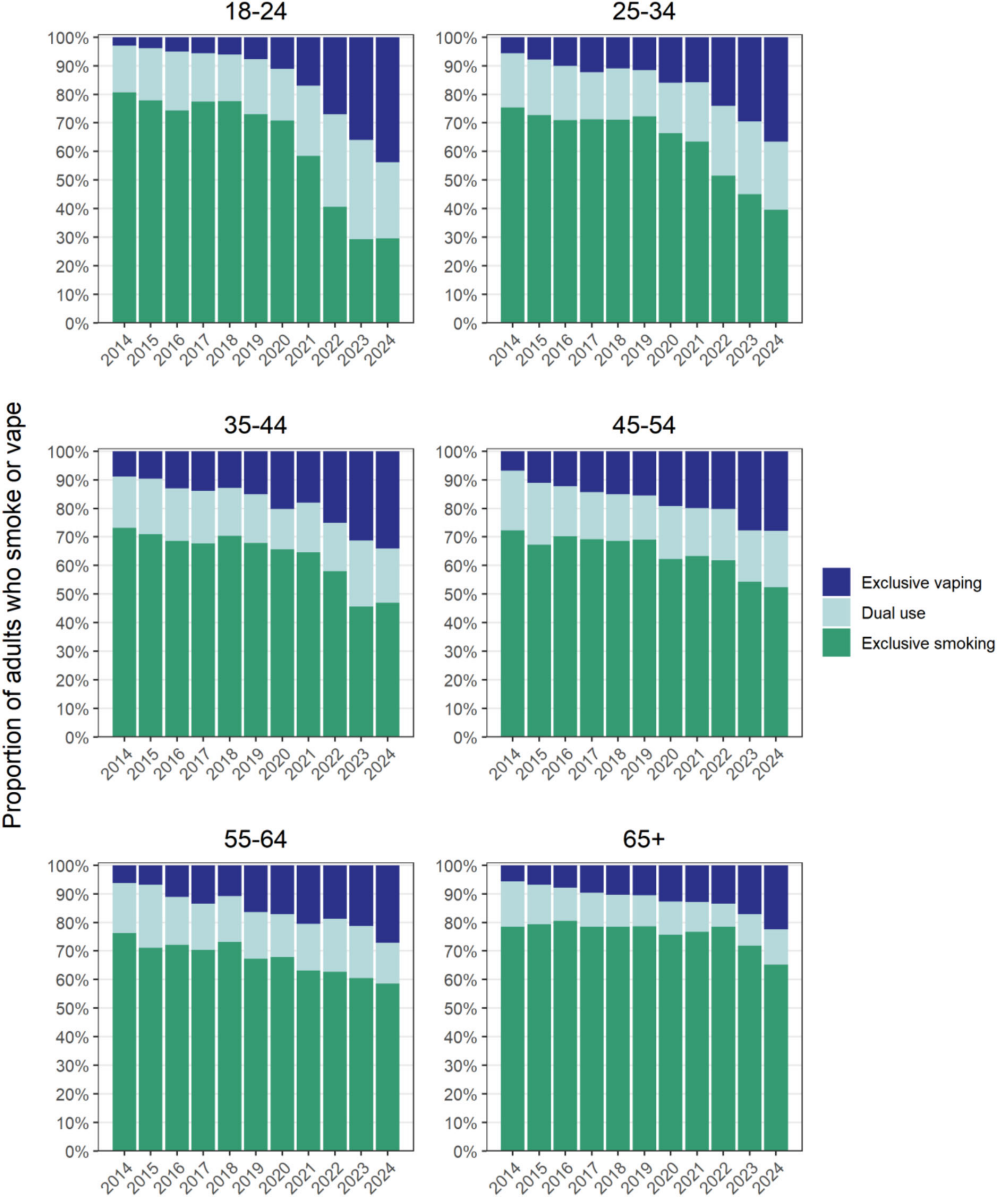
Prevalence of exclusive smoking, exclusive vaping and dual use among adults who smoke or vape in England, 2014 to 2024. Estimates with 95% CI are provided in Data S3, along with corresponding figures and estimates for all adults (not just those who smoke or vape).

Disaggregating inhaled nicotine use into exclusive smoking, exclusive vaping and dual use revealed further differences across age groups (Figure 2, Tables 1, 2, Data S3). Among 18 to 24‐year‐olds who smoked or vaped, the proportion who exclusively smoked fell sharply [from 80.7% in 2014 to 29.6% in 2024; PR = 0.37 (0.32–0.42)], especially after 2020. Exclusive vaping increased in parallel [from 3.0% to 43.7%; PR = 14.6 (9.66–25.3)], becoming the most common pattern of inhaled nicotine use in this age group. Dual use also increased, but to a lesser extent (from 16.3% to 26.7%; PR = 1.64 (1.34–2.02)]. Among all 18 to 24‐year‐olds (i.e. not just those who smoked and vaped), the absolute prevalence of exclusive smoking fell from 21.0% to 10.5%, the prevalence of exclusive vaping increased from 0.8% to 15.5% and the prevalence of dual use increased from 4.3% to 9.5% (Data S3).

Adults age 25 to 34 years old followed a similar trajectory, with substantial growth in exclusive vaping [from 5.6% to 36.6%; PR = 6.54 (4.94–8.98)] alongside a decline in exclusive smoking [from 75.3% to 39.5%; PR = 0.53 (0.48–0.58)]. In the 35 to 44 years and 45 to 54 years age groups, exclusive smoking remained the predominant pattern of use throughout the period (46.9% and 52.4%, respectively, in 2024). However, exclusive vaping increased gradually [from 8.9% to 34.1%; PR = 3.83 (2.98–5.29) and from 6.8% to 27.9%; PR = 4.10 (3.03–5.73), respectively], especially from 2021 onward. In the older groups (55–64 and ≥65 years old), exclusive smoking remained dominant (58.5% and 65.2%, respectively, in 2024), but there was still a notable increase in exclusive vaping over time [e.g. from 5.7% in 2014 to 22.5% in 2024 among those ≥65 years old; PR = 3.95 (2.79–5.82)], with relatively limited dual use in the oldest group (≥65 years) compared with other age groups.

### Smoking type and pattern among adults who smoke

**FIGURE 3:**
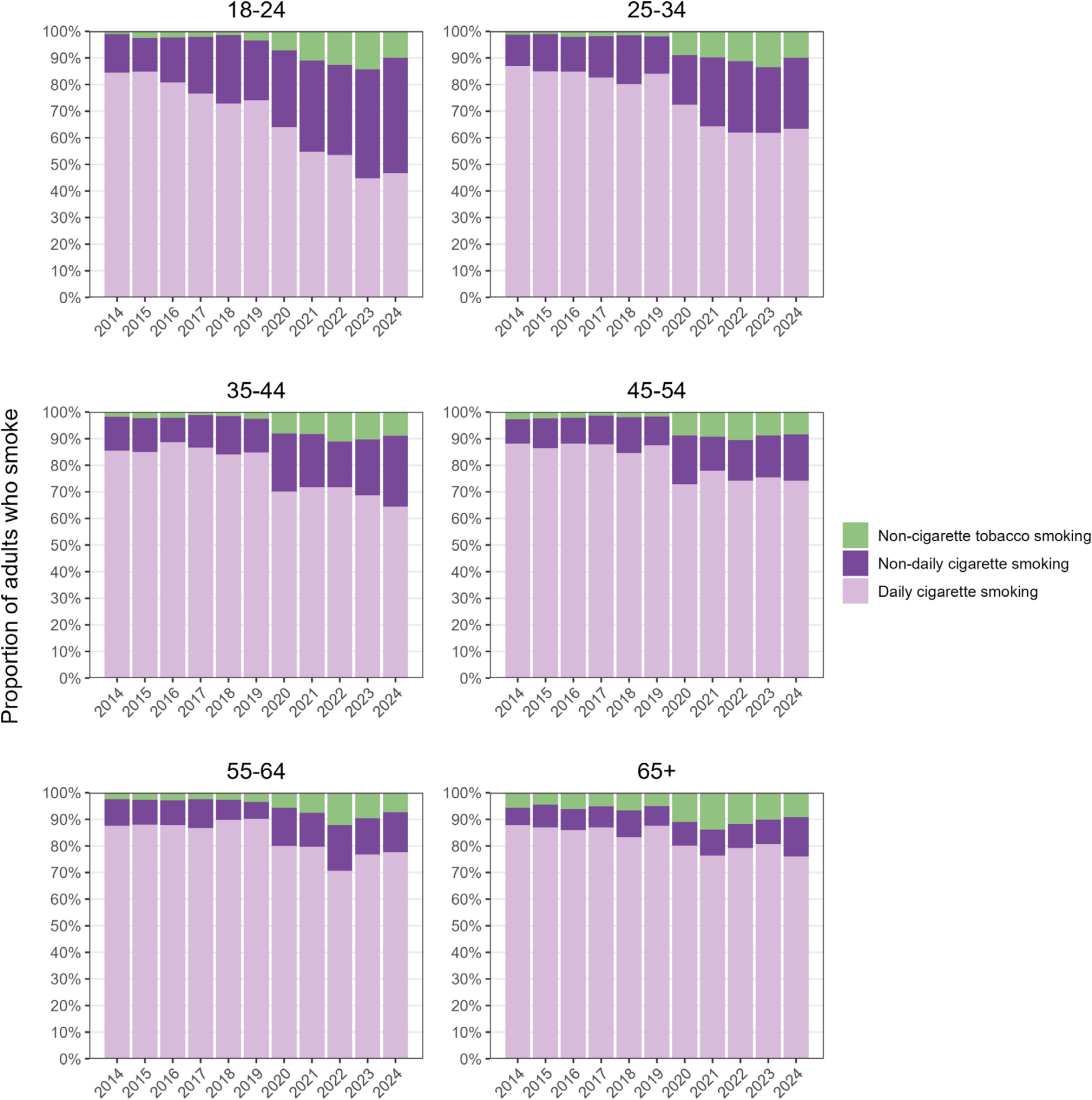
Smoking type and pattern among adults who smoke in England, 2014 to 2024. Estimates with 95% CI are provided in Data S4, along with corresponding figures and estimates for all adults (not just those who smoke).

As the prevalence of smoking declined, there were also shifts in the type and pattern of tobacco use among adults who smoked (Figure 3, Tables 1, 2, Data S4). In 2014, across all ages, the majority who smoked reported smoking cigarettes daily (range = 84.5%–88.1%). However, among 18 to 24‐year‐olds who smoked, the proportion smoking cigarettes daily declined sharply [from 84.5% in 2014 to 46.7% in 2024; PR = 0.55 (0.50–0.62)], particularly after 2020, while non‐daily cigarette smoking increased [from 14.5% to 43.3%; PR = 2.99 (2.43–3.74)]. By 2023, fewer than half (44.8%) of 18‐ to 24‐year‐olds who smoked reported daily cigarette smoking. The proportion who did not smoke cigarettes at all, but smoked other forms of tobacco increased substantially between 2019 and 2023 (from 3.5% to 14.4%), then uncertainly declined (to 9.9%) in 2024. Among all 18 to 24‐year‐olds (i.e. not just those who smoked), the absolute prevalence of daily cigarette smoking decreased from 21.4% to 9.3% between 2014 and 2024, while non‐daily cigarette smoking increased from 3.7% to 8.6% and exclusive non‐cigarette tobacco smoking increased from 0.3% to 2.0% (Data S4).

Patterns were similar among adults 25 to 44 years old, with a gradual decline in daily smoking accompanied by increases in both non‐daily cigarette smoking and non‐cigarette tobacco smoking. However, these changes were less pronounced than among the youngest group. In 2024, approximately two‐thirds of those who smoked reported smoking cigarettes daily (63.4% and 64.4% among those age 25–34 years and 35–44 years, respectively).

In the older (45–54 years, 55–64 years and ≥65 years) age groups, daily cigarette smoking remained the predominant pattern throughout the study period, with only modest increases in non‐cigarette tobacco smoking and especially non‐daily smoking. Compared with younger adults, these groups showed more stability in smoking behaviour, with slower transitions away from daily cigarette use. In 2024, approximately three‐quarters who smoked still smoked cigarettes daily (74.2%, 77.7% and 76.1% among those age 45–54 years, 55–64 years and ≥65 years, respectively). Declines in the absolute prevalence of daily smoking between 2014 and 2024 were ever more modest with increasing age (Data S4).

### Smoking history among adults who vape

**FIGURE 4:**
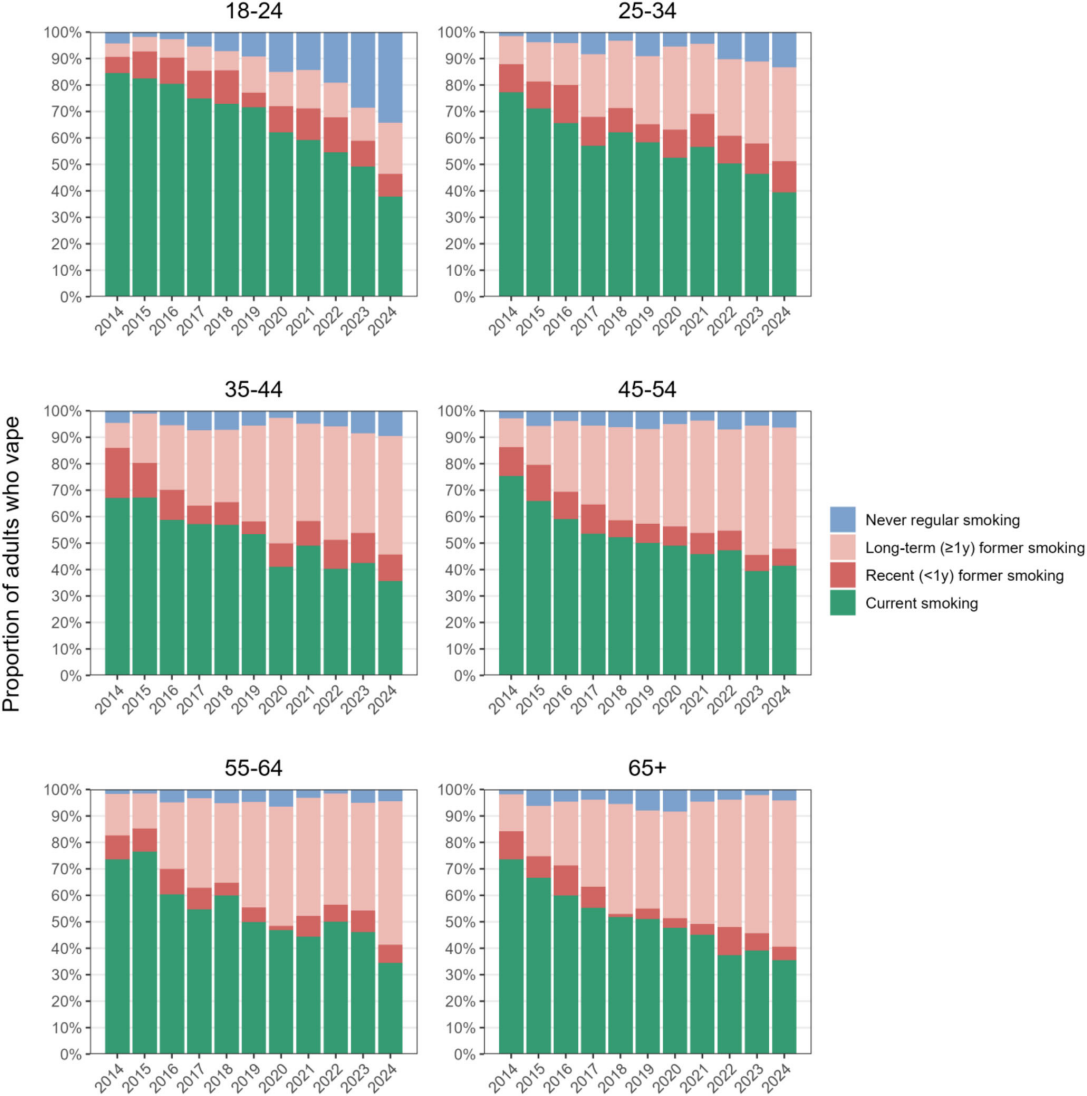
Smoking history among adults who vape in England, 2014 to 2024. Estimates with 95% CI are provided in Data S5.

As the prevalence of vaping increased, there were changes in the composition of the vaping population in terms of their smoking history (Figure 4, Tables 1, 2, Data S5). Across all age groups and years, the majority of adults who vaped had a history of smoking. However, the proportion of those who vaped who reported current smoking declined steadily from 2014 to 2024 in all age groups.

Among 18‐ to 24‐year‐olds who vaped, this was largely driven by an increase in the proportion who had never regularly smoked [from 4.3% in 2014 to 34.3% in 2024; PR = 7.98 (4.56–26.2); +30.0, (24.8–35.6) percentage points), with this subgroup growing to a similar size as those who reported current smoking by 2024 (37.9%). There was also an increase, albeit smaller, in the proportion who reported long‐term former smoking [i.e. quit ≥1 year ago; from 5.1% to 19.3%; PR = 3.78 (1.82–12.4); +14.2 (8.02–19.5) percentage points].

Among those age 25 to 34 years old who vaped, a similar pattern was observed, but with a smaller absolute increase in the proportion who had never smoked [from 1.5% to 13.3%; +11.8 (8.69–14.9) percentage points] and a larger absolute increase in the proportion who reported long‐term former smoking [from 10.7% to 35.5%; +24.9 (18.9–30.9) percentage points].

In the older age groups (35–44 years, 45–54 years, 55–64 years and ≥65 years) who vaped, the decline in the proportion who currently smoked was mostly offset by increases in the proportion who reported long‐term former smoking. However, there still appeared to be increases in the proportion who had never regularly smoked, even in the oldest age group (≥65; from 1.9% to 4.2%), although the 95% CIs around this change indicate some uncertainty (+2.29 (−2.27 to 6.97) percentage points].

## DISCUSSION

This study presents a comprehensive picture of changing patterns of nicotine use among adults of different ages in England between 2014 and 2024, revealing substantial generational shifts.

### Prevalence of use of different nicotine products among adults

The most striking changes occurred among younger adults (<45 years) where daily smoking declined markedly, particularly among 18‐ to 24‐year‐olds. Meanwhile, vaping increased sharply and surpassed smoking by 2023 in 18‐ to 24‐year‐olds. This coincided with rising overall nicotine use, driven by growth in exclusive vaping and, to a lesser extent, dual use and nicotine pouch uptake. Use of heated tobacco products remained rare. Similar trends were observed among adults 25 to 44 years old, although changes were smaller with increasing age. These shifts likely reflect the growing appeal and availability of novel nicotine products, in particular e‐cigarettes and nicotine pouches, which have been marketed in ways that particularly appeal to younger consumers, for example, endorsement by social media influencers, highlighting a range of appealing flavours, modern product designs and lifestyle appeal [23, 24]. Although younger adults did not initially show higher uptake of vaping than older age groups, recent trends suggest that these products have become increasingly embedded in youth culture [23, 25].

Changes in nicotine use were less pronounced among adults 45 to 54 years old. While vaping prevalence increased and smoking declined, these shifts did not lead to large changes in overall nicotine use. Exclusive smoking remained the dominant pattern of use throughout, and the majority of those who smoked in this age group continued to report daily cigarette use. Adults ≥55 years old exhibited even more stability in nicotine use patterns, with smaller increases in vaping and an uncertain decline in smoking. This relative stability may reflect longer smoking histories, higher levels of nicotine dependence and greater resistance to behaviour change (including less interest in trying new products) among older adults [15, 26, 27]. The way e‐cigarettes are marketed and portrayed may also mean they are less appealing to older age groups [28]. Alternatively, self‐selection pressures (such as healthy survivor effects), resulting in a more homogenous group less motivated to quit smoking, may contribute to the relative lack of change in this age group. Nevertheless, even in the oldest age group, vaping prevalence increased and exclusive vaping became more common, indicating gradual but ongoing changes in older age groups.

### Smoking type and pattern among adults who smoke

Across all age groups, daily smoking prevalence declined more sharply than the prevalence of smoking overall. This pattern was particularly pronounced among young adults (18–24 years), among whom daily smoking prevalence fell to below 10% and non‐daily smoking became the predominant pattern among those who smoked. This indicates a meaningful reduction in the most harmful form of nicotine consumption [3].

Looking at longer‐term trends, our recent analysis of Smoking Toolkit Study data from 2007 to 2024 shows that adult smoking prevalence has declined fairly steadily over the past two decades [29]. Although this suggests that the rise in vaping has not markedly accelerated the overall decline in smoking, the present data show recent reductions have been largest among young adults, who have had the highest uptake of vaping. Our previous analysis also showed that until around 2020, most smoking among adults was daily cigarette smoking [29]. Since then, trends have diverged, with sharp reductions in daily smoking coinciding with the rapid rise in vaping, a shift that has been particularly pronounced among young adults. Together, these data suggest that vaping has not only displaced smoking for many young adults, but may also have contributed to changes in the pattern and intensity of tobacco use among those who continue to smoke (i.e. dual users). Recent data show there has been a shift away from more regular smoking toward more regular vaping since 2016 among adults who both smoke and vape, and that daily vaping with non‐daily smoking is a more common pattern of dual use among those who are younger [30]. These shifts may also potentially reflect changes in social norms, whereby smoking—particularly daily smoking—has become less visible and less socially acceptable in younger cohorts, while vaping has gained traction as the normative form of nicotine use [31].

A further consideration is the observed increase in non‐cigarette tobacco smoking since 2020, particularly among younger adults. This may reflect product substitution driven by differences in UK tobacco regulations across product categories. Although cigarettes have been subject to standardised packaging and minimum pack sizes since May 2017 and a ban on menthol and other flavours since May 2020, these restrictions did not extend to products such as cigars, cigarillos or pipe tobacco. As a result, non‐cigarette tobacco products can still be sold in colourful, branded packaging, in smaller and cheaper pack sizes and with flavour options (including menthol) that remain appealing to younger consumers [32, 33, 34]. The tobacco industry has capitalised on these regulatory gaps, for example, by introducing flavoured cigarillos that closely resemble menthol cigarettes around the time the menthol ban was implemented [35, 36]. Importantly, if declines in cigarette use are partly offset by substitution with other combustible tobacco products, then reductions in cigarette smoking prevalence may overstate progress in reducing tobacco‐related harm [29]. Although the absolute prevalence of non‐cigarette tobacco use remained low compared with cigarettes, the upward trend highlights the need for comprehensive policies that cover all combustible tobacco products to avoid undermining public health gains.

### Smoking history among adults who vape

Another notable finding was the evolving smoking history profile of adults who vape. Across all age groups, the proportion of adults using e‐cigarettes who reported former tobacco smoking increased, indicating that e‐cigarettes are being used as a tool for successful smoking cessation. Most people do not take up vaping spontaneously after quitting smoking, rather, many people continue vaping for prolonged periods after using e‐cigarettes to stop smoking [37, 38]. Consequently, as more people have successfully quit smoking with e‐cigarettes the share of adults who vape who report former smoking has grown.

Although most vapers continued to have a history of smoking, there were also increases in the proportion who had never regularly smoked, particularly in the youngest age group. By 2024, more than one in three 18‐ to 24‐year‐olds who vaped had never regularly smoked. This suggests that a growing number of young adults are initiating nicotine use with vaping, rather than transitioning from smoking. This shift likely reflects both displacement of smoking initiation in younger generations and the growing appeal of vaping as a standalone behaviour, culturally distinct from smoking.

It is probable that some people who vape, but have never regularly smoked may have otherwise initiated regular smoking in the absence of vaping [6], which would confer greater health risks [3]. However, with the overall prevalence of nicotine use increasing to levels not seen since the 1990s [39], there will also be some young adults taking up vaping who would not have otherwise smoked [5, 6]. This will expose them to more risk than if they had not used any nicotine products at all [3]. The emergence of newer products, such as e‐cigarettes and nicotine pouches, may contribute to this trend, because they offer a less harmful alternative to traditional smoking [3, 26] and are more socially acceptable [40], cheaper and more discreet [41, 42]. These features may make them attractive not only to people who smoke and seek alternatives, but also to individuals who may never have otherwise used nicotine. Although these products may help reduce smoking rates, they also create new entry points for nicotine use, potentially broadening the user base and driving up overall prevalence even as smoking declines. The presence of people who vape, but have never regularly smoked in older age groups raises important questions about how and why people are entering the nicotine market later in life. These individuals may provide a clearer view of new recruitment into nicotine use, given they were teens and young adults (the periods of life when smoking initiation is most common) [43] in the pre‐vaping era.

### Implications

The policy implications of these trends in nicotine use will depend on the ultimate goal of regulation. Traditionally, tobacco control efforts have focused on reducing tobacco‐related harm, with an emphasis on lowering smoking prevalence and its associated health risks [44, 45]. However, the recent rapid growth in e‐cigarette use and the emergence of other alternative nicotine products have led to the suggestion of a broader objective: a nicotine‐free society [46, 47]. Our findings suggest such a goal requires nuanced consideration. Although vaping appears to be playing a role in accelerating declines in daily cigarette smoking—especially among young adults—it is also contributing to a rise in overall nicotine use. This dual effect underscores the complexity of the evolving nicotine landscape and suggests policies must strike a balance between minimising harm and preventing new uptake. Given e‐cigarettes expose users to much lower numbers and levels of toxicants compared to combustible tobacco [3], a continued shift in nicotine use from smoking toward vaping and other less harmful products would likely yield a net public health benefit [48] —even if the total number of nicotine users increases. By reducing exposure to the most dangerous forms of nicotine consumption [3], such a shift could lead to meaningful reductions in smoking‐related disease and mortality at the population level. In this case, prioritising harm reduction over reducing uptake of nicotine may be the most effective strategy for safeguarding public health. However, if these novel products confer a risk reduction that is counterbalanced by an increase in prevalence of use, then there could be population harms.

Current patterns of nicotine use in England present a unique opportunity in the history of tobacco control, with a large number of adults who smoke non‐daily already using alternative nicotine products. This group could be well‐positioned to quit smoking entirely, but widespread misperceptions about the relative harms of smoking and alternatives [49] may hinder smoking cessation efforts [50]. Public health messaging that clearly communicates the relative harms of smoking and alternative nicotine products is crucial for enabling people to make informed decisions about the products they use.

Although this study focuses exclusively on England, it is important to note that smoking and vaping trends may differ internationally. For example, declines in smoking among young adults in the United States have been more rapid than those observed in England [51]. The reason is not clear, but differences may reflect variations in regulatory approaches (e.g. an increase in age of sale from 18 to 21 in the United States) and public attitudes toward smoking. Although a detailed cross‐country comparison is beyond the scope of this paper, such comparisons could provide valuable insights into how context shapes nicotine use trends.

### Strengths and limitations

This study has several strengths. It uses large‐scale, nationally representative data collected consistently over a decade, capturing a period of substantial change in nicotine use. Disaggregation by product type and smoking history provides a more detailed understanding of user profiles.

However, there were also limitations. Although the observed progression of nicotine use patterns across age groups is suggestive of a cohort effect, our analysis cannot definitively separate age, period and cohort influences. Formal age‐period‐cohort modelling was not feasible at this stage because many of the trends we documented have unfolded within a short time frame, but may be a useful avenue for future studies over the longer‐term. Despite the large sample, 95% CIs were wide for estimates of rarer outcomes. The cross‐sectional design means causal relationships cannot be established. For example, while increases in vaping coincided with declines in smoking, the extent to which vaping directly contributed to these changes or whether other factors (e.g. tobacco control policies, shifts in social norms) played a more significant role remains unclear. In addition, the repeat cross‐sectional design meant we could not assess within‐person transitions over time, such as whether dual users ultimately quit smoking. The survey did not assess vaping history, so it was not possible to analyse differences in smoking patterns between those who currently vaped and those who had vaped in the past and quit. Finally, we did not explore the underlying motivations or reasons for changes in nicotine use behaviours, such as the influence of peer groups, marketing or public health policies. Understanding these drivers would offer valuable insights for policymakers looking to design interventions that are responsive to changing patterns of nicotine use.

## CONCLUSIONS

Together, these patterns suggest a rapidly changing nicotine landscape in England. Vaping is increasingly decoupled from smoking, particularly among younger and emerging middle‐age adults, and may continue to rise in prevalence among those who have never regularly smoked tobacco as current cohorts age. If current trends persist, and absent significant policy change, the population‐level burden of daily cigarette smoking may continue to decline as younger cohorts— already exhibiting low daily cigarette smoking rates—age into older adulthood. The delayed, but visible rise in vaping among those who have never regularly smoked across successive age bands hints at a future nicotine market that is less dominated by combustibles—a public health improvement if the overall nicotine user base also shrinks. However, if the total user base expands, the net impact becomes more complex, depending on the balance between the reduction in harm from fewer people smoking and the potential risks faced by individuals who would not otherwise have used nicotine products. This evolving landscape may be influenced by ongoing legislative efforts, such as the Tobacco and Vapes Bill. Understanding these trends is critical for informing future policy, enabling it to respond effectively to the evolving dynamics of nicotine consumption.

## AUTHOR CONTRIBUTIONS

**Sarah E. Jackson:** Conceptualization (lead); formal analysis (lead); investigation (equal); methodology (equal); visualization (lead); writing—original draft (lead); writing—review and editing (equal). **Lion Shahab:** Conceptualization (supporting); funding acquisition (equal); investigation (equal); methodology (equal); writing—review and editing (equal). **Vera Buss:** Data curation (lead); investigation (equal); writing—review and editing (equal). **Harry Tattan‐Birch:** Investigation (equal); writing—review and editing (equal). **Sharon Cox:** Investigation (equal); writing—review and editing (equal). **Eve Taylor:** Investigation (equal); writing—review and editing (equal). **Jamie Brown:** Conceptualization (supporting); data curation (supporting); funding acquisition (equal); investigation (equal); methodology (equal); supervision (lead); writing—review and editing (equal).

## DECLARATION OF INTERESTS

J.B. has received (most recently in 2018) unrestricted research funding from Pfizer and J&J, who manufacture smoking cessation medications. L.S. has received honoraria for talks, unrestricted research grants and travel expenses to attend meetings and workshops from manufactures of smoking cessation medications (Pfizer; J&J), and has acted as paid reviewer for grant awarding bodies and as a paid consultant for health care companies. All authors declare no financial links with tobacco companies, e‐cigarette manufacturers or their representatives.

## ETHICS APPROVAL

Ethical approval for the STS was granted originally by the UCL Ethics Committee (ID 0498/001). Participants provide informed consent to take part in the study, and all methods are carried out in accordance with relevant regulations. The data are not collected by UCL and are anonymised when received by UCL.

## Supporting information


**Data S1.** Supplementary Information.

**Data S2.** Supplementary Information.

**Data S3.** Supplementary Information.

**Data S4.** Supplementary Information.

**Data S5.** Supplementary Information.

## Data Availability

Data are available on Open Science Framework (https://osf.io/wf6eu).
