# Supplementary material for: The changing face of nicotine use in England: Age‐specific annual trends, 2014 to 2024
Source: Addiction. 2025 Dec 7;121(3):549–63. doi: 10.1111/add.70243 (PMC12887924; doi:10.1111/add.70243)
Supplement: Supplementary file 1 — Data S1. Supplementary Information. [file ADD-121-549-s004.pdf]

Supplementary File 1: Unweighted sample sizes

Table S1. Unweighted sample sizes by age and year

	Age group (years)					
	**18-24**	**25-34**	**35-44**	**45-54**	**55-64**	**≥65**
Adults						
2014	2979	3052	2944	2989	3011	4770
2015	2784	3099	2938	2971	3075	4725
2016	2632	2963	2881	3104	3193	5281
2017	2557	2819	2839	3052	3215	5553
2018	2653	2893	2814	3099	3292	5651
2019	2853	3015	2983	2896	3099	5534
2020	1930	2535	2454	2993	3249	5196
2021	2008	2843	2718	3604	3265	5310
2022	2315	3115	2870	3344	3103	4665
2023	2074	3039	3068	3377	3458	4844
2024	2175	3174	3078	3111	3376	4934
Adults who smoke or vape						
2014	777	841	690	699	582	528
2015	758	839	695	715	619	527
2016	682	771	666	730	595	597
2017	638	747	610	684	580	551
2018	612	809	653	703	577	593
2019	596	778	597	599	564	539
2020	502	710	557	524	486	472
2021	554	830	589	634	523	549
2022	699	965	682	672	451	471
2023	672	925	746	729	686	523
2024	730	990	768	636	618	523

Table continues on next page.

Supplementary File 1: Unweighted sample sizes

Table S1. *Continued*

	Age group (years)					
	**18-24**	**25-34**	**35-44**	**45-54**	**55-64**	**≥65**
Adults who smoke						
2014	756	799	634	654	549	497
2015	733	781	631	642	580	494
2016	647	697	589	646	533	549
2017	600	658	530	590	504	497
2018	577	723	574	608	512	529
2019	556	692	515	501	473	483
2020	439	587	439	418	399	414
2021	458	697	486	509	414	480
2022	510	733	512	527	366	404
2023	433	647	514	527	537	434
2024	424	620	493	451	443	409
Adults who vape						
2014	153	198	179	190	129	110
2015	165	227	197	221	167	97
2016	172	216	199	214	166	120
2017	142	214	201	209	169	121
2018	136	238	189	218	161	129
2019	169	221	181	184	182	116
2020	156	246	193	194	162	113
2021	229	304	205	227	193	129
2022	414	465	287	269	162	106
2023	480	510	397	333	273	145
2024	511	596	420	309	263	178
