# Supplementary material for: The changing face of nicotine use in England: Age‐specific annual trends, 2014 to 2024
Source: Addiction. 2025 Dec 7;121(3):549–63. doi: 10.1111/add.70243 (PMC12887924; doi:10.1111/add.70243)
Supplement: Supplementary file 2 — Data S2. Supplementary Information. [file ADD-121-549-s001.pdf]

Supplementary File 2: Prevalence of nicotine use, smoking, vaping, heated tobacco use, and nicotine pouch use

Table S2. Nicotine use prevalence by age group and year

	Nicotine use, % [95% confidence interval]										
	**2014**	**2015**	**2016**	**2017**	**2018**	**2019**	**2020**	**2021**	**2022**	**2023**	**2024**
18-24	26.1	27.5	26.2	25.6	23.8	20.9	27.2	28.6	30.7	34.8	36.5
	[24.4–27.8]	[25.7–29.4]	[24.5–28.0]	[23.8–27.4]	[22.0–25.5]	[19.2–22.5]	[25.0–29.4]	[26.5–30.8]	[28.7–32.8]	[32.5–37.0]	[34.2–38.8]
25-34	27.0	27.0	26.2	27.2	27.9	26.3	28.7	30.5	33.0	32.2	35.1
	[25.4–28.7]	[25.3–28.8]	[24.5–27.9]	[25.4–29.0]	[26.1–29.6]	[24.6–28.0]	[26.8–30.6]	[28.6–32.3]	[31.1–34.8]	[30.4–34.0]	[33.3–37.0]
35-44	22.5	23.8	23.4	21.7	22.9	19.9	23.7	22.1	25.4	25.8	27.2
	[20.9–24.1]	[22.2–25.5]	[21.7–25.0]	[20.1–23.3]	[21.3–24.6]	[18.3–21.4]	[21.9–25.6]	[20.4–23.8]	[23.6–27.2]	[24.1–27.5]	[25.4–28.9]
45-54	21.7	22.1	22.5	22.1	21.7	19.9	17.7	18.0	21.8	22.9	22.0
	[20.2–23.2]	[20.5–23.7]	[21.0–24.0]	[20.5–23.6]	[20.2–23.2]	[18.4–21.4]	[16.3–19.2]	[16.7–19.4]	[20.3–23.4]	[21.4–24.5]	[20.4–23.6]
55-64	17.9	19.0	18.3	18.1	16.9	17.4	15.7	16.0	14.8	20.6	19.6
	[16.5–19.3]	[17.5–20.4]	[16.9–19.7]	[16.7–19.5]	[15.6–18.3]	[16.0–18.9]	[14.4–17.1]	[14.7–17.4]	[13.5–16.2]	[19.2–22.1]	[18.1–21.1]
≥65	10.1	10.1	10.8	9.6	10.4	9.4	9.1	10.8	10.6	11.1	11.2
	[9.3–11.0]	[9.2–11.0]	[9.9–11.7]	[8.8–10.4]	[9.6–11.2]	[8.6–10.2]	[8.3–9.9]	[9.9–11.7]	[9.6–11.5]	[10.1–12.0]	[10.2–12.1]

Table S3. Smoking prevalence by age group and year

	Smoking, % [95% confidence interval]										
	**2014**	**2015**	**2016**	**2017**	**2018**	**2019**	**2020**	**2021**	**2022**	**2023**	**2024**
18-24	25.3	26.5	24.9	24.1	22.3	19.2	24.2	23.6	22.1	21.9	19.9
	[23.6–26.9]	[24.7–28.3]	[23.2–26.7]	[22.4–25.9]	[20.6–24.1]	[17.6–20.8]	[22.0–26.3]	[21.6–25.7]	[20.2–24.0]	[19.9–23.8]	[18.1–21.8]
25-34	25.5	24.9	23.6	23.8	24.8	23.3	24.0	25.4	24.8	22.4	21.7
	[23.9–27.2]	[23.2–26.6]	[21.9–25.2]	[22.2–25.5]	[23.1–26.5]	[21.6–24.9]	[22.2–25.8]	[23.6–27.1]	[23.1–26.5]	[20.8–24.0]	[20.0–23.3]
35-44	20.5	21.6	20.4	18.6	20.0	16.9	18.9	17.9	19.0	17.5	17.6
	[19.0–22.0]	[19.9–23.2]	[18.8–21.9]	[17.1–20.2]	[18.4–21.5]	[15.4–18.3]	[17.2–20.6]	[16.4–19.5]	[17.4–20.6]	[16.1–19.0]	[16.1–19.2]
45-54	20.2	19.6	19.8	18.9	18.4	16.7	14.2	14.3	17.3	16.5	15.7
	[18.7–21.7]	[18.1–21.1]	[18.3–21.2]	[17.4–20.3]	[17.0–19.8]	[15.3–18.1]	[12.8–15.5]	[13.1–15.6]	[15.9–18.8]	[15.1–17.9]	[14.2–17.1]
55-64	16.8	17.7	16.3	15.6	15.1	14.6	13.1	12.7	12.0	16.1	14.2
	[15.4–18.2]	[16.2–19.1]	[15.0–17.6]	[14.3–16.9]	[13.8–16.4]	[13.3–15.9]	[11.8–14.3]	[11.5–13.9]	[10.8–13.2]	[14.7–17.4]	[12.9–15.6]
≥65	9.6	9.4	10.0	8.7	9.3	8.4	7.9	9.3	9.1	9.1	8.6
	[8.7–10.4]	[8.5–10.3]	[9.1–10.8]	[7.9–9.5]	[8.5–10.1]	[7.6–9.2]	[7.2–8.7]	[8.5–10.2]	[8.2–10.1]	[8.3–10.0]	[7.8–9.5]

Supplementary File 2: Prevalence of nicotine use, smoking, vaping, heated tobacco use, and nicotine pouch use

Table S4. Vaping prevalence by age group and year

	**Vaping, % [95% confidence interval]**										
	**2014**	**2015**	**2016**	**2017**	**2018**	**2019**	**2020**	**2021**	**2022**	**2023**	**2024**
18-24	5.0	6.1	6.7	5.8	5.3	5.6	8.0	11.9	18.0	24.1	25.0
	[4.2–5.9]	[5.1–7.0]	[5.7–7.8]	[4.8–6.8]	[4.4–6.3]	[4.7–6.5]	[6.7–9.2]	[10.3–13.4]	[16.3–19.7]	[22.1–26.2]	[22.9–27.0]
25-34	6.7	7.4	7.6	7.8	8.1	7.3	9.6	11.0	15.8	17.4	20.7
	[5.7–7.6]	[6.4–8.4]	[6.6–8.7]	[6.8–8.9]	[7.0–9.1]	[6.3–8.3]	[8.4–10.8]	[9.8–12.3]	[14.4–17.3]	[16.0–18.9]	[19.1–22.3]
35-44	6.0	6.9	7.4	7.0	6.8	6.4	8.1	7.7	10.6	13.9	14.2
	[5.1–7.0]	[5.9–8.0]	[6.3–8.4]	[6.0–8.0]	[5.8–7.8]	[5.4–7.4]	[7.0–9.3]	[6.6–8.8]	[9.4–11.9]	[12.5–15.3]	[12.8–15.6]
45-54	6.0	7.2	6.7	6.8	6.8	6.1	6.6	6.6	8.3	10.5	10.3
	[5.1–6.9]	[6.2–8.2]	[5.8–7.6]	[5.9–7.7]	[5.9–7.7]	[5.2–7.0]	[5.7–7.6]	[5.7–7.5]	[7.3–9.3]	[9.3–11.6]	[9.2–11.5]
55-64	4.3	5.5	5.1	5.3	4.5	5.7	5.1	5.9	5.5	8.0	8.1
	[3.5–5.0]	[4.6–6.3]	[4.3–5.9]	[4.5–6.2]	[3.8–5.3]	[4.8–6.6]	[4.3–5.9]	[5.0–6.8]	[4.6–6.4]	[7.1–9.0]	[7.1–9.1]
≥65	2.2	2.1	2.1	2.1	2.2	2.0	2.2	2.5	2.3	3.1	3.9
	[1.7–2.6]	[1.6–2.5]	[1.7–2.5]	[1.7–2.5]	[1.8–2.6]	[1.6–2.4]	[1.8–2.6]	[2.1–3.0]	[1.8–2.7]	[2.6–3.6]	[3.3–4.5]

Table S5. Heated tobacco use prevalence by age group and year

	**Heated tobacco use, % [95% confidence interval]**										
	**2014**	**2015**	**2016**	**2017**	**2018**	**2019**	**2020**	**2021**	**2022**	**2023**	**2024**
18-24	-	-	-	0.2	0.0	0.1	0.1	0.6	0.9	0.4	0.4
				[0.0–0.3]	[0.0–0.0]	[0.0–0.3]	[0.0–0.2]	[0.2–1.0]	[0.4–1.4]	[0.1–0.7]	[0.1–0.8]
25-34	-	-	-	0.1	0.3	0.1	0.2	0.6	0.4	0.3	0.5
				[0.0–0.2]	[0.1–0.5]	[0.0–0.2]	[0.0–0.4]	[0.3–0.9]	[0.2–0.6]	[0.1–0.5]	[0.3–0.8]
35-44	-	-	-	0.2	0.1	0.1	0.2	0.4	0.2	0.3	0.4
				[0.1–0.4]	[0.0–0.3]	[0.0–0.2]	[0.0–0.3]	[0.1–0.6]	[0.0–0.4]	[0.1–0.5]	[0.2–0.6]
45-54	-	-	-	0.1	0.1	0.2	0.3	0.2	0.3	0.3	0.1
				[0.0–0.2]	[0.0–0.2]	[0.0–0.4]	[0.1–0.5]	[0.1–0.3]	[0.1–0.5]	[0.1–0.5]	[0.0–0.2]
55-64	-	-	-	0.1	0.1	0.0	0.2	0.1	0.1	0.2	0.2
				[0.0–0.2]	[0.0–0.2]	[0.0–0.1]	[0.0–0.3]	[0.0–0.3]	[0.0–0.3]	[0.1–0.4]	[0.0–0.3]
≥65	-	-	-	0.0	0.1	0.1	0.0	0.1	0.1	0.1	0.1
				[0.0–0.1]	[0.0–0.2]	[0.0–0.1]	[0.0–0.1]	[0.0–0.1]	[0.0–0.1]	[0.0–0.1]	[0.0–0.2]

Supplementary File 2: Prevalence of nicotine use, smoking, vaping, heated tobacco use, and nicotine pouch use

Table S6. Nicotine pouch use prevalence by age group and year

	Nicotine pouch use, % [95% confidence interval]										
	**2014**	**2015**	**2016**	**2017**	**2018**	**2019**	**2020**	**2021**	**2022**	**2023**	**2024**
18-24	-	-	-	-	-	-	-	0.7 [0.3–1.2]	0.9 [0.5–1.4]	1.8 [1.2–2.5]	3.4 [2.6–4.3]
25-34	-	-	-	-	-	-	-	0.5 [0.2–0.7]	0.6 [0.3–0.8]	0.8 [0.4–1.2]	1.3 [0.9–1.7]
35-44	-	-	-	-	-	-	-	0.5 [0.2–0.8]	0.3 [0.1–0.4]	0.3 [0.1–0.6]	0.6 [0.3–0.8]
45-54	-	-	-	-	-	-	-	0.2 [0.0–0.4]	0.5 [0.2–0.7]	0.3 [0.1–0.6]	0.7 [0.3–1.0]
55-64	-	-	-	-	-	-	-	0.1 [0.0–0.3]	0.1 [0.0–0.2]	0.2 [0.1–0.4]	0.1 [0.0–0.3]
≥65	-	-	-	-	-	-	-	0.0 [0.0–0.1]	0.0 [0.0–0.1]	0.1 [0.0–0.2]	0.1 [0.0–0.2]
