# Supplementary material for: The changing face of nicotine use in England: Age‐specific annual trends, 2014 to 2024
Source: Addiction. 2025 Dec 7;121(3):549–63. doi: 10.1111/add.70243 (PMC12887924; doi:10.1111/add.70243)
Supplement: Supplementary file 3 — Data S3. Supplementary Information. [file ADD-121-549-s005.pdf]

Supplementary File 3: Patterns of exclusive and dual use of smoking and vaping

Table S7. Exclusive smoking prevalence among adults who smoke or vape, by age group and year

	Exclusive smoking, % [95% confidence interval]										
	**2014**	**2015**	**2016**	**2017**	**2018**	**2019**	**2020**	**2021**	**2022**	**2023**	**2024**
18-24	80.7	77.9	74.3	77.4	77.6	73.0	70.8	58.4	40.6	29.3	29.6
	[77.8–83.6]	[74.7–81.1]	[70.9–77.8]	[74.0–80.9]	[74.0–81.2]	[69.1–77.0]	[66.5–75.0]	[53.9–62.9]	[36.6–44.6]	[25.5–33.1]	[25.9–33.2]
25-34	75.3	72.7	70.9	71.3	71.0	72.3	66.4	63.4	51.5	45.1	39.5
	[72.2–78.4]	[69.4–76.0]	[67.4–74.4]	[67.8–74.7]	[67.7–74.3]	[68.9–75.7]	[62.7–70.1]	[59.9–67.0]	[48.1–55.0]	[41.6–48.5]	[36.2–42.9]
35-44	73.1	70.9	68.6	67.7	70.3	67.8	65.7	64.7	58.0	45.6	46.9
	[69.5–76.7]	[67.2–74.6]	[64.7–72.4]	[63.9–71.6]	[66.6–74.0]	[63.6–72.0]	[61.5–69.9]	[60.5–68.8]	[53.9–62.1]	[41.7–49.4]	[43.0–50.8]
45-54	72.3	67.3	70.2	69.2	68.6	69.0	62.2	63.3	61.7	54.2	52.4
	[68.7–75.8]	[63.5–71.1]	[66.7–73.6]	[65.5–72.8]	[64.9–72.2]	[65.0–73.0]	[57.8–66.7]	[59.3–67.4]	[57.7–65.7]	[50.4–58.1]	[48.2–56.7]
55-64	76.2	71.2	72.1	70.4	73.2	67.3	67.9	63.1	62.6	60.5	58.5
	[72.3–80.1]	[67.2–75.1]	[68.3–75.9]	[66.5–74.3]	[69.4–77.0]	[63.1–71.5]	[63.5–72.3]	[58.7–67.5]	[57.8–67.5]	[56.6–64.5]	[54.2–62.8]
≥65	78.5	79.4	80.5	78.4	78.5	78.6	75.7	76.7	78.5	71.8	65.2
	[74.7–82.3]	[75.4–83.4]	[77.2–83.8]	[74.8–82.0]	[75.0–81.9]	[74.9–82.4]	[71.6–79.8]	[72.9–80.4]	[74.6–82.5]	[67.7–75.9]	[60.6–69.7]

Table S8. Exclusive vaping prevalence among adults who smoke or vape, by age group and year

	Exclusive vaping, % [95% confidence interval]										
	**2014**	**2015**	**2016**	**2017**	**2018**	**2019**	**2020**	**2021**	**2022**	**2023**	**2024**
18-24	3.0	3.9	5.0	5.7	6.1	7.7	11.1	17.0	27.0	36.0	43.7
	[1.7–4.3]	[2.2–5.5]	[3.3–6.7]	[3.8–7.6]	[4.0–8.2]	[5.1–10.2]	[8.3–13.9]	[13.6–20.4]	[23.4–30.6]	[32.0–39.9]	[39.8–47.7]
25-34	5.6	7.9	10.0	12.3	11.0	11.5	16.0	15.9	24.0	29.4	36.6
	[3.9–7.3]	[5.8–10.0]	[7.7–12.4]	[9.8–14.9]	[8.7–13.3]	[9.0–14.1]	[13.2–18.7]	[13.2–18.5]	[21.1–27.0]	[26.3–32.6]	[33.3–39.9]
35-44	8.9	9.6	13.0	13.8	12.8	15.0	20.3	18.0	25.1	31.3	34.1
	[6.5–11.2]	[7.1–12.0]	[10.1–15.8]	[10.9–16.8]	[10.0–15.6]	[11.7–18.3]	[16.8–23.8]	[14.7–21.4]	[21.5–28.6]	[27.7–34.9]	[30.5–37.7]
45-54	6.8	11.2	12.2	14.3	15.0	15.5	19.2	19.9	20.2	27.7	27.9
	[4.8–8.9]	[8.5–13.8]	[9.7–14.8]	[11.6–17.1]	[12.1–18.0]	[12.5–18.5]	[15.7–22.7]	[16.5–23.2]	[16.9–23.5]	[24.2–31.2]	[24.1–31.6]
55-64	6.3	6.8	11.0	13.4	10.7	16.4	17.1	20.5	18.7	21.2	27.2
	[4.0–8.6]	[4.6–9.0]	[8.3–13.8]	[10.5–16.3]	[8.1–13.3]	[13.0–19.8]	[13.6–20.6]	[16.8–24.2]	[14.8–22.5]	[18.0–24.5]	[23.4–31.0]
≥65	5.7	6.9	7.8	9.7	10.4	10.4	12.7	12.8	13.5	17.2	22.5
	[3.6–7.7]	[4.4–9.4]	[5.6–10.0]	[7.1–12.3]	[7.8–12.9]	[7.7–13.2]	[9.5–15.9]	[9.8–15.8]	[10.2–16.8]	[13.7–20.6]	[18.5–26.4]

Supplementary File 3: Patterns of exclusive and dual use of smoking and vaping

Table S9. Dual use prevalence among adults who smoke or vape, by age group and year

	**Dual use of smoking and vaping, % [95% confidence interval]**										
	**2014**	**2015**	**2016**	**2017**	**2018**	**2019**	**2020**	**2021**	**2022**	**2023**	**2024**
18-24	16.3	18.2	20.7	16.9	16.3	19.3	18.2	24.6	32.4	34.8	26.7
	[13.6–19.0]	[15.3–21.1]	[17.5–23.9]	[13.8–20.0]	[13.2–19.5]	[15.9–22.7]	[14.5–21.8]	[20.7–28.6]	[28.6–36.2]	[30.9–38.6]	[23.2–30.1]
25-34	19.1	19.4	19.1	16.4	18.0	16.2	17.7	20.7	24.4	25.5	23.9
	[16.3–21.9]	[16.5–22.3]	[16.0–22.1]	[13.6–19.2]	[15.2–20.7]	[13.5–18.9]	[14.6–20.7]	[17.7–23.7]	[21.4–27.4]	[22.4–28.6]	[20.8–26.9]
35-44	18.0	19.6	18.5	18.4	16.9	17.2	14.1	17.3	17.0	23.1	18.9
	[14.9–21.1]	[16.3–22.8]	[15.3–21.6]	[15.3–21.6]	[13.9–19.9]	[13.8–20.6]	[10.9–17.2]	[14.0–20.5]	[14.0–19.9]	[19.8–26.4]	[15.9–22.0]
45-54	20.9	21.6	17.6	16.5	16.4	15.5	18.5	16.8	18.1	18.0	19.7
	[17.7–24.1]	[18.2–24.9]	[14.8–20.5]	[13.6–19.4]	[13.6–19.2]	[12.3–18.8]	[14.9–22.2]	[13.6–20.0]	[14.9–21.3]	[15.1–21.0]	[16.3–23.1]
55-64	17.5	22.1	16.9	16.2	16.0	16.3	15.0	16.4	18.7	18.2	14.3
	[14.1–21.0]	[18.4–25.7]	[13.7–20.0]	[13.0–19.4]	[12.9–19.2]	[13.0–19.6]	[11.7–18.3]	[12.9–19.8]	[14.6–22.8]	[15.1–21.3]	[11.2–17.4]
≥65	15.8	13.7	11.7	11.9	11.1	10.9	11.6	10.5	8.0	11.0	12.4
	[12.4–19.2]	[10.3–17.2]	[9.0–14.4]	[9.1–14.8]	[8.5–13.8]	[8.0–13.8]	[8.6–14.7]	[7.9–13.2]	[5.5–10.6]	[8.1–13.9]	[9.2–15.5]

Supplementary File 3: Patterns of exclusive and dual use of smoking and vaping

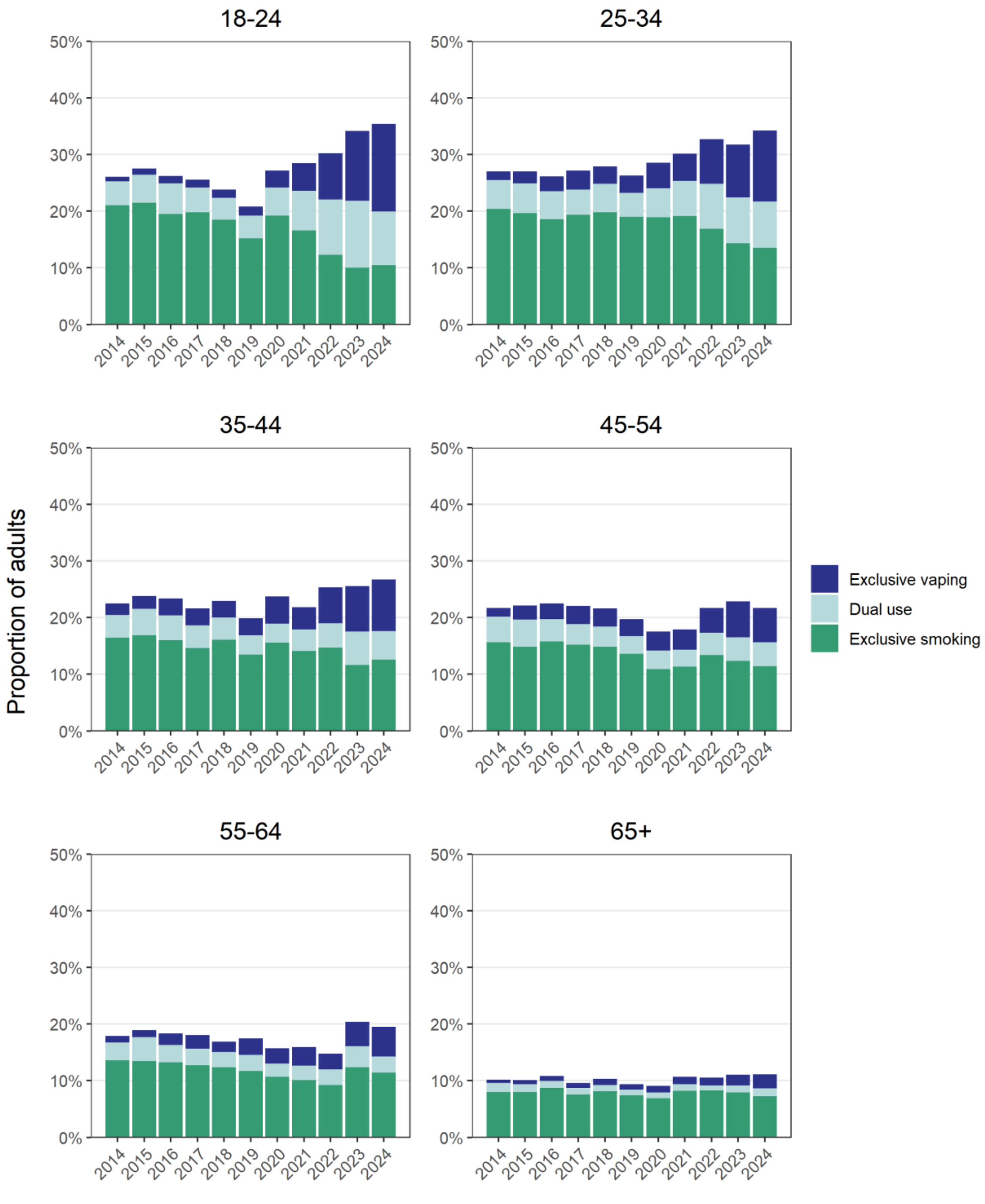

Figure S1. Prevalence of exclusive smoking, exclusive vaping, and dual use among adults in England, 2014 to 2024. Estimates with 95% confidence intervals are provided in **Tables S9-11**.

Supplementary File 3: Patterns of exclusive and dual use of smoking and vaping

Table S10. Exclusive smoking prevalence among adults, by age group and year

	Exclusive smoking, % [95% confidence interval]										
	**2014**	**2015**	**2016**	**2017**	**2018**	**2019**	**2020**	**2021**	**2022**	**2023**	**2024**
18-24	21.0	21.5	19.5	19.8	18.5	15.2	19.2	16.6	12.3	10.0	10.5
	[19.5–22.6]	[19.8–23.1]	[17.9–21.1]	[18.2–21.5]	[16.9–20.0]	[13.8–16.7]	[17.3–21.2]	[14.8–18.4]	[10.8–13.8]	[8.5–11.5]	[9.0–11.9]
25-34	20.4	19.7	18.6	19.4	19.8	19.0	19.0	19.1	16.9	14.3	13.5
	[18.9–21.9]	[18.1–21.2]	[17.1–20.0]	[17.8–21.0]	[18.2–21.4]	[17.5–20.5]	[17.3–20.6]	[17.5–20.7]	[15.4–18.3]	[12.9–15.7]	[12.2–14.9]
35-44	16.4	16.9	16.0	14.7	16.1	13.5	15.6	14.1	14.7	11.6	12.6
	[15.0–17.8]	[15.4–18.4]	[14.6–17.5]	[13.3–16.0]	[14.7–17.5]	[12.1–14.8]	[14.0–17.1]	[12.7–15.5]	[13.2–16.2]	[10.4–12.9]	[11.2–13.9]
45-54	15.7	14.9	15.8	15.2	14.8	13.6	10.9	11.3	13.4	12.4	11.4
	[14.3–17.0]	[13.6–16.2]	[14.5–17.1]	[13.9–16.6]	[13.6–16.1]	[12.3–15.0]	[9.7–12.1]	[10.2–12.4]	[12.1–14.7]	[11.2–13.6]	[10.1–12.7]
55-64	13.6	13.5	13.2	12.7	12.4	11.7	10.7	10.1	9.2	12.4	11.4
	[12.4–14.9]	[12.2–14.8]	[12.0–14.4]	[11.5–13.9]	[11.2–13.6]	[10.5–12.9]	[9.5–11.9]	[9.0–11.2]	[8.1–10.3]	[11.2–13.5]	[10.2–12.7]
≥65	8.0	8.0	8.7	7.5	8.1	7.4	6.9	8.2	8.3	7.9	7.3
	[7.2–8.8]	[7.2–8.8]	[7.9–9.5]	[6.8–8.3]	[7.4–8.9]	[6.7–8.1]	[6.2–7.6]	[7.4–9.0]	[7.4–9.2]	[7.1–8.7]	[6.5–8.1]

Table S11. Exclusive vaping prevalence among adults, by age group and year

	Exclusive vaping, % [95% confidence interval]										
	**2014**	**2015**	**2016**	**2017**	**2018**	**2019**	**2020**	**2021**	**2022**	**2023**	**2024**
18-24	0.8	1.1	1.3	1.4	1.4	1.6	3.0	4.8	8.2	12.3	15.5
	[0.4–1.1]	[0.6–1.5]	[0.9–1.8]	[1.0–1.9]	[0.9–2.0]	[1.0–2.2]	[2.2–3.8]	[3.8–5.9]	[6.9–9.4]	[10.7–13.9]	[13.8–17.2]
25-34	1.5	2.1	2.6	3.4	3.1	3.0	4.6	4.8	7.9	9.3	12.5
	[1.0–2.0]	[1.5–2.7]	[2.0–3.3]	[2.6–4.1]	[2.4–3.7]	[2.3–3.7]	[3.7–5.4]	[3.9–5.6]	[6.8–8.9]	[8.2–10.5]	[11.2–13.8]
35-44	2.0	2.3	3.0	3.0	2.9	3.0	4.8	3.9	6.3	8.0	9.1
	[1.4–2.5]	[1.7–2.9]	[2.3–3.7]	[2.3–3.7]	[2.3–3.6]	[2.3–3.7]	[3.9–5.7]	[3.1–4.7]	[5.3–7.4]	[6.9–9.1]	[8.0–10.2]
45-54	1.5	2.5	2.8	3.2	3.3	3.1	3.4	3.6	4.4	6.3	6.1
	[1.0–1.9]	[1.9–3.1]	[2.2–3.4]	[2.5–3.8]	[2.6–3.9]	[2.4–3.7]	[2.7–4.0]	[2.9–4.2]	[3.6–5.2]	[5.4–7.2]	[5.1–7.0]
55-64	1.1	1.3	2.0	2.4	1.8	2.9	2.7	3.3	2.8	4.3	5.3
	[0.7–1.5]	[0.9–1.7]	[1.5–2.5]	[1.9–3.0]	[1.4–2.3]	[2.2–3.5]	[2.1–3.3]	[2.6–3.9]	[2.1–3.4]	[3.6–5.1]	[4.5–6.2]
≥65	0.6	0.7	0.8	0.9	1.1	1.0	1.2	1.4	1.4	1.9	2.5
	[0.4–0.8]	[0.4–1.0]	[0.6–1.1]	[0.7–1.2]	[0.8–1.3]	[0.7–1.3]	[0.8–1.5]	[1.0–1.7]	[1.1–1.8]	[1.5–2.3]	[2.0–3.0]

Supplementary File 3: Patterns of exclusive and dual use of smoking and vaping

Table S12. Dual use prevalence among adults, by age group and year

	Dual use of smoking and vaping, % [95% confidence interval]										
	**2014**	**2015**	**2016**	**2017**	**2018**	**2019**	**2020**	**2021**	**2022**	**2023**	**2024**
18-24	4.3	5.0	5.4	4.3	3.9	4.0	4.9	7.0	9.8	11.9	9.5
	[3.5–5.0]	[4.1–5.9]	[4.5–6.3]	[3.5–5.2]	[3.1–4.7]	[3.3–4.8]	[3.9–6.0]	[5.8–8.3]	[8.5–11.1]	[10.3–13.4]	[8.1–10.8]
25-34	5.2	5.2	5.0	4.5	5.0	4.3	5.1	6.2	8.0	8.1	8.2
	[4.3–6.0]	[4.4–6.1]	[4.1–5.9]	[3.7–5.3]	[4.2–5.8]	[3.5–5.0]	[4.1–6.0]	[5.2–7.2]	[6.9–9.1]	[7.0–9.2]	[7.0–9.3]
35-44	4.1	4.7	4.3	4.0	3.9	3.4	3.3	3.8	4.3	5.9	5.1
	[3.3–4.8]	[3.8–5.5]	[3.5–5.1]	[3.3–4.7]	[3.1–4.6]	[2.7–4.1]	[2.5–4.1]	[3.0–4.5]	[3.5–5.1]	[5.0–6.8]	[4.2–6.0]
45-54	4.5	4.8	4.0	3.6	3.6	3.1	3.3	3.0	3.9	4.1	4.3
	[3.8–5.3]	[3.9–5.6]	[3.3–4.7]	[3.0–4.3]	[2.9–4.2]	[2.4–3.8]	[2.5–4.0]	[2.4–3.6]	[3.2–4.7]	[3.4–4.8]	[3.5–5.1]
55-64	3.1	4.2	3.1	2.9	2.7	2.8	2.4	2.6	2.8	3.7	2.8
	[2.5–3.8]	[3.4–5.0]	[2.5–3.7]	[2.3–3.5]	[2.1–3.3]	[2.2–3.5]	[1.8–2.9]	[2.0–3.2]	[2.1–3.4]	[3.0–4.4]	[2.2–3.4]
≥65	1.6	1.4	1.3	1.1	1.2	1.0	1.1	1.1	0.8	1.2	1.4
	[1.2–2.0]	[1.0–1.8]	[1.0–1.6]	[0.9–1.4]	[0.9–1.4]	[0.7–1.3]	[0.8–1.4]	[0.8–1.4]	[0.6–1.1]	[0.9–1.6]	[1.0–1.8]
