# Supplementary material for: The changing face of nicotine use in England: Age‐specific annual trends, 2014 to 2024
Source: Addiction. 2025 Dec 7;121(3):549–63. doi: 10.1111/add.70243 (PMC12887924; doi:10.1111/add.70243)
Supplement: Supplementary file 4 — Data S4. Supplementary Information. [file ADD-121-549-s003.pdf]

Supplementary File 4: Smoking type and pattern

Table S13. Proportion who smoke cigarettes daily among adults who smoke, by age group and year

	Daily cigarette smoking, % [95% confidence interval]										
	**2014**	**2015**	**2016**	**2017**	**2018**	**2019**	**2020**	**2021**	**2022**	**2023**	**2024**
18-24	84.5	84.8	80.7	76.6	72.8	74.0	64.1	54.7	53.6	44.8	46.7
	[81.7–87.2]	[82.0–87.5]	[77.6–83.8]	[73.1–80.1]	[69.0–76.6]	[70.1–77.9]	[59.1–69.0]	[49.7–59.7]	[48.8–58.3]	[39.7–49.9]	[41.5–52.0]
25-34	86.9	85.0	84.8	82.6	80.1	84.0	72.4	64.4	61.9	61.9	63.4
	[84.4–89.5]	[82.1–87.9]	[82.1–87.6]	[79.6–85.7]	[77.0–83.2]	[81.1–87.0]	[68.5–76.3]	[60.5–68.2]	[58.1–65.8]	[57.9–65.9]	[59.3–67.6]
35-44	85.6	84.9	88.7	86.7	84.1	84.8	70.1	71.7	71.7	68.7	64.4
	[82.6–88.5]	[81.7–88.2]	[85.9–91.4]	[83.6–89.8]	[81.0–87.3]	[81.4–88.2]	[65.5–74.7]	[67.4–75.9]	[67.5–75.9]	[64.5–72.8]	[59.8–69.0]
45-54	88.1	86.4	88.1	87.8	84.7	87.6	72.9	78.0	74.2	75.6	74.2
	[85.4–90.8]	[83.3–89.4]	[85.5–90.7]	[85.1–90.6]	[81.7–87.7]	[84.6–90.7]	[68.4–77.5]	[74.2–81.8]	[70.3–78.2]	[71.8–79.3]	[69.8–78.7]
55-64	87.5	88.0	87.9	86.7	89.7	90.2	80.0	79.7	70.7	76.7	77.7
	[84.4–90.6]	[84.9–91.0]	[85.0–90.9]	[83.6–89.8]	[86.9–92.6]	[87.3–93.1]	[75.7–84.2]	[75.6–83.9]	[65.7–75.7]	[73.0–80.5]	[73.5–81.8]
≥65	87.9	87.0	85.9	87.0	83.4	87.6	80.2	76.4	79.3	80.7	76.1
	[84.7–91.0]	[83.8–90.3]	[82.9–89.0]	[83.9–90.0]	[80.1–86.7]	[84.4–90.8]	[76.2–84.2]	[72.4–80.4]	[75.1–83.6]	[76.9–84.6]	[71.8–80.4]

Table S14. Proportion who smoke cigarettes non-daily among adults who smoke, by age group and year

	Non-daily cigarette smoking, % [95% confidence interval]										
	**2014**	**2015**	**2016**	**2017**	**2018**	**2019**	**2020**	**2021**	**2022**	**2023**	**2024**
18-24	14.5	12.7	17.1	21.3	25.7	22.5	28.8	34.4	33.9	40.8	43.3
	[11.8–17.2]	[10.2–15.2]	[14.2–20.1]	[17.9–24.6]	[22.0–29.5]	[18.9–26.1]	[24.1–33.5]	[29.6–39.2]	[29.4–38.4]	[35.8–45.8]	[38.1–48.5]
25-34	11.9	13.9	13.0	15.6	18.3	13.9	18.7	25.8	26.9	24.7	26.5
	[9.4–14.3]	[11.1–16.8]	[10.4–15.6]	[12.6–18.5]	[15.3–21.4]	[11.2–16.7]	[15.4–22.1]	[22.3–29.4]	[23.3–30.4]	[21.2–28.3]	[22.8–30.3]
35-44	12.6	12.8	9.2	12.2	14.4	12.6	21.8	20.1	17.1	21.1	26.6
	[9.8–15.5]	[9.8–15.8]	[6.8–11.6]	[9.2–15.2]	[11.3–17.4]	[9.6–15.7]	[17.7–25.9]	[16.3–24.0]	[13.7–20.5]	[17.5–24.7]	[22.4–30.9]
45-54	9.2	11.2	9.8	10.7	13.3	10.7	18.3	12.8	15.2	15.6	17.4
	[6.7–11.6]	[8.3–14.0]	[7.4–12.1]	[8.2–13.3]	[10.4–16.2]	[7.8–13.5]	[14.3–22.3]	[9.8–15.8]	[11.9–18.4]	[12.5–18.7]	[13.6–21.3]
55-64	10.1	9.3	9.3	10.9	7.5	6.3	14.5	12.8	17.1	13.7	15.0
	[7.2–13.0]	[6.6–12.0]	[6.7–11.9]	[8.1–13.7]	[5.1–10.0]	[3.9–8.7]	[10.7–18.2]	[9.3–16.3]	[13.1–21.1]	[10.6–16.7]	[11.4–18.6]
≥65	6.5	8.5	7.9	7.9	10.0	7.4	8.8	9.8	9.0	9.2	14.6
	[4.0–9.0]	[5.7–11.3]	[5.5–10.4]	[5.4–10.4]	[7.3–12.7]	[4.9–10.0]	[6.0–11.5]	[6.8–12.7]	[5.9–12.1]	[6.4–12.0]	[10.9–18.3]

Supplementary File 4: Smoking type and pattern

Table S15. Proportion who exclusively smoke non-cigarette tobacco among adults who smoke, by age group and year

	Exclusive non-cigarette smoking, % [95% confidence interval]										
	**2014**	**2015**	**2016**	**2017**	**2018**	**2019**	**2020**	**2021**	**2022**	**2023**	**2024**
18-24	1.0	2.5	2.2	2.1	1.5	3.5	7.2	10.9	12.5	14.4	9.9
	[0.3–1.8]	[1.3–3.8]	[1.0–3.4]	[0.9–3.3]	[0.5–2.4]	[1.6–5.3]	[4.6–9.7]	[7.9–14.0]	[9.5–15.6]	[10.8–17.9]	[7.0–12.9]
25-34	1.2	1.1	2.1	1.8	1.6	2.0	8.9	9.8	11.2	13.4	10.0
	[0.4–2.0]	[0.4–1.8]	[1.1–3.2]	[0.7–2.9]	[0.6–2.5]	[0.9–3.1]	[6.3–11.4]	[7.5–12.2]	[8.8–13.7]	[10.7–16.1]	[7.4–12.6]
35-44	1.8	2.2	2.1	1.1	1.5	2.6	8.1	8.2	11.2	10.2	9.0
	[0.7–2.9]	[0.8–3.7]	[0.8–3.5]	[0.2–2.0]	[0.5–2.5]	[0.9–4.2]	[5.2–11.0]	[5.8–10.7]	[8.2–14.1]	[7.5–12.9]	[6.4–11.5]
45-54	2.7	2.4	2.1	1.4	2.0	1.7	8.8	9.2	10.6	8.9	8.3
	[1.4–4.1]	[1.1–3.8]	[0.9–3.3]	[0.4–2.4]	[0.9–3.1]	[0.5–3.0]	[5.9–11.7]	[6.5–11.9]	[7.9–13.3]	[6.3–11.4]	[5.5–11.1]
55-64	2.4	2.7	2.8	2.4	2.7	3.5	5.5	7.4	12.2	9.6	7.4
	[1.0–3.7]	[1.1–4.4]	[1.3–4.2]	[0.9–3.9]	[1.3–4.2]	[1.6–5.3]	[3.1–8.0]	[4.8–10.0]	[8.4–15.9]	[7.1–12.1]	[4.9–9.8]
≥65	5.6	4.4	6.1	5.1	6.6	5.0	11.0	13.8	11.7	10.1	9.3
	[3.6–7.6]	[2.5–6.4]	[4.1–8.2]	[3.2–7.0]	[4.4–8.7]	[2.9–7.0]	[7.8–14.2]	[10.7–17.0]	[8.4–15.0]	[7.1–13.0]	[6.6–12.0]

Supplementary File 4: Smoking type and pattern

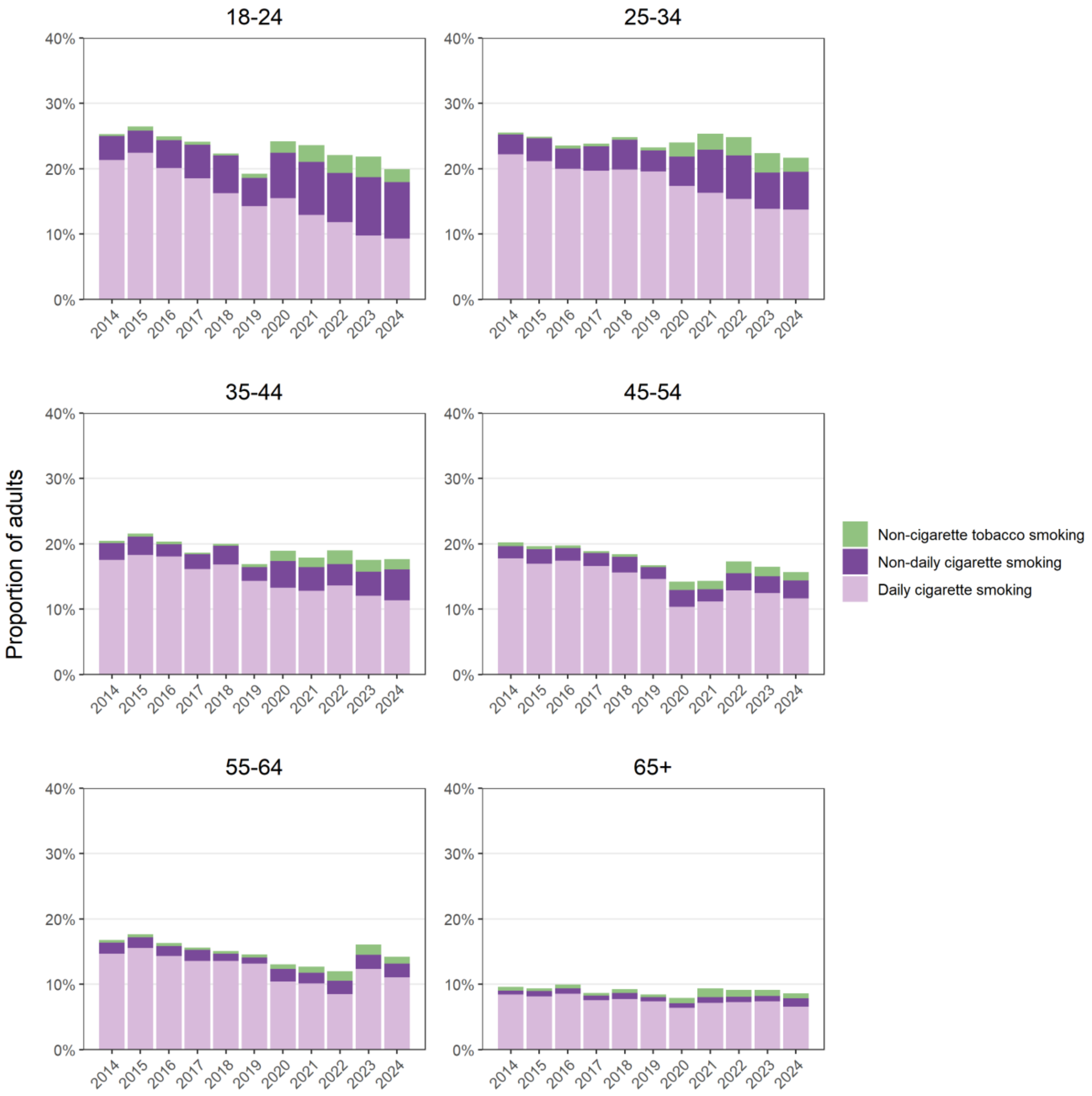

Figure S2. Smoking type and pattern among adults in England, 2014 to 2024. Estimates with 95% confidence intervals are provided in **Tables S15-17.**

Supplementary File 4: Smoking type and pattern

Table S16. Proportion who smoke cigarettes daily among adults, by age group and year

	Daily cigarette smoking, % [95% confidence interval]										
	**2014**	**2015**	**2016**	**2017**	**2018**	**2019**	**2020**	**2021**	**2022**	**2023**	**2024**
18-24	21.4	22.4	20.1	18.5	16.3	14.2	15.5	12.9	11.8	9.8	9.3
	[19.8–22.9]	[20.8–24.1]	[18.5–21.7]	[16.9–20.1]	[14.7–17.8]	[12.8–15.7]	[13.7–17.3]	[11.3–14.5]	[10.4–13.3]	[8.4–11.2]	[7.9–10.7]
25-34	22.2	21.2	20.0	19.7	19.9	19.6	17.4	16.3	15.4	13.9	13.8
	[20.6–23.7]	[19.6–22.7]	[18.5–21.5]	[18.1–21.3]	[18.3–21.4]	[18.0–21.1]	[15.8–19.0]	[14.8–17.8]	[14.0–16.8]	[12.5–15.2]	[12.3–15.2]
35-44	17.5	18.3	18.1	16.2	16.8	14.3	13.3	12.8	13.6	12.0	11.4
	[16.1–19.0]	[16.8–19.8]	[16.6–19.5]	[14.7–17.6]	[15.4–18.3]	[12.9–15.7]	[11.8–14.7]	[11.5–14.2]	[12.2–15.0]	[10.8–13.3]	[10.0–12.7]
45-54	17.8	17.0	17.4	16.6	15.6	14.6	10.3	11.2	12.9	12.5	11.6
	[16.4–19.2]	[15.6–18.4]	[16.0–18.8]	[15.2–17.9]	[14.3–16.9]	[13.3–16.0]	[9.2–11.5]	[10.1–12.3]	[11.5–14.2]	[11.2–13.7]	[10.4–12.9]
55-64	14.7	15.5	14.3	13.6	13.5	13.2	10.4	10.1	8.5	12.3	11.1
	[13.4–16.0]	[14.2–16.9]	[13.1–15.6]	[12.3–14.8]	[12.3–14.8]	[11.9–14.4]	[9.3–11.6]	[9.0–11.2]	[7.4–9.5]	[11.1–13.5]	[9.8–12.3]
≥65	8.4	8.2	8.6	7.6	7.7	7.4	6.4	7.1	7.2	7.4	6.6
	[7.6–9.2]	[7.4–9.0]	[7.8–9.3]	[6.8–8.3]	[7.0–8.5]	[6.6–8.1]	[5.7–7.1]	[6.4–7.9]	[6.4–8.1]	[6.6–8.2]	[5.8–7.4]

Table S17. Proportion who smoke cigarettes non-daily among adults, by age group and year

	Non-daily cigarette smoking, % [95% confidence interval]										
	**2014**	**2015**	**2016**	**2017**	**2018**	**2019**	**2020**	**2021**	**2022**	**2023**	**2024**
18-24	3.7	3.4	4.3	5.1	5.7	4.3	7.0	8.1	7.5	8.9	8.6
	[3.0–4.4]	[2.7–4.1]	[3.5–5.1]	[4.3–6.0]	[4.8–6.7]	[3.6–5.1]	[5.7–8.3]	[6.8–9.5]	[6.3–8.7]	[7.6–10.3]	[7.3–10.0]
25-34	3.0	3.5	3.1	3.7	4.5	3.2	4.5	6.6	6.7	5.5	5.8
	[2.4–3.7]	[2.7–4.2]	[2.4–3.7]	[3.0–4.5]	[3.7–5.4]	[2.6–3.9]	[3.6–5.4]	[5.5–7.6]	[5.7–7.7]	[4.7–6.4]	[4.9–6.7]
35-44	2.6	2.8	1.9	2.3	2.9	2.1	4.1	3.6	3.3	3.7	4.7
	[2.0–3.2]	[2.1–3.4]	[1.4–2.4]	[1.7–2.9]	[2.2–3.5]	[1.6–2.7]	[3.3–5.0]	[2.8–4.4]	[2.6–3.9]	[3.0–4.4]	[3.9–5.5]
45-54	1.8	2.2	1.9	2.0	2.4	1.8	2.6	1.8	2.6	2.6	2.7
	[1.3–2.4]	[1.6–2.8]	[1.4–2.4]	[1.5–2.5]	[1.9–3.0]	[1.3–2.3]	[2.0–3.2]	[1.4–2.3]	[2.0–3.2]	[2.0–3.1]	[2.1–3.4]
55-64	1.7	1.6	1.5	1.7	1.1	0.9	1.9	1.6	2.1	2.2	2.1
	[1.2–2.2]	[1.2–2.1]	[1.1–2.0]	[1.2–2.2]	[0.7–1.5]	[0.6–1.3]	[1.4–2.4]	[1.2–2.1]	[1.5–2.6]	[1.7–2.7]	[1.6–2.7]
≥65	0.6	0.8	0.8	0.7	0.9	0.6	0.7	0.9	0.8	0.8	1.3
	[0.4–0.9]	[0.5–1.1]	[0.5–1.0]	[0.5–0.9]	[0.7–1.2]	[0.4–0.8]	[0.5–0.9]	[0.6–1.2]	[0.5–1.1]	[0.6–1.1]	[0.9–1.6]

Supplementary File 4: Smoking type and pattern

Table S18. Proportion who exclusively smoke non-cigarette tobacco among adults, by age group and year

	Exclusive non-cigarette smoking, % [95% confidence interval]										
	**2014**	**2015**	**2016**	**2017**	**2018**	**2019**	**2020**	**2021**	**2022**	**2023**	**2024**
18-24	0.3	0.7	0.5	0.5	0.3	0.7	1.7	2.6	2.8	3.1	2.0
	[0.1–0.4]	[0.3–1.0]	[0.3–0.8]	[0.2–0.8]	[0.1–0.5]	[0.3–1.0]	[1.1–2.4]	[1.8–3.3]	[2.1–3.5]	[2.3–4.0]	[1.4–2.6]
25-34	0.3	0.3	0.5	0.4	0.4	0.5	2.1	2.5	2.8	3.0	2.2
	[0.1–0.5]	[0.1–0.4]	[0.2–0.8]	[0.2–0.7]	[0.2–0.6]	[0.2–0.7]	[1.5–2.8]	[1.9–3.1]	[2.2–3.4]	[2.4–3.6]	[1.6–2.8]
35-44	0.4	0.5	0.4	0.2	0.3	0.4	1.5	1.5	2.1	1.8	1.6
	[0.1–0.6]	[0.2–0.8]	[0.2–0.7]	[0.0–0.4]	[0.1–0.5]	[0.2–0.7]	[1.0–2.1]	[1.0–1.9]	[1.5–2.7]	[1.3–2.3]	[1.1–2.0]
45-54	0.6	0.5	0.4	0.3	0.4	0.3	1.2	1.3	1.8	1.5	1.3
	[0.3–0.8]	[0.2–0.7]	[0.2–0.7]	[0.1–0.5]	[0.2–0.6]	[0.1–0.5]	[0.8–1.7]	[0.9–1.7]	[1.3–2.3]	[1.0–1.9]	[0.8–1.8]
55-64	0.4	0.5	0.5	0.4	0.4	0.5	0.7	0.9	1.5	1.5	1.0
	[0.2–0.6]	[0.2–0.8]	[0.2–0.7]	[0.1–0.6]	[0.2–0.6]	[0.2–0.8]	[0.4–1.1]	[0.6–1.3]	[1.0–1.9]	[1.1–2.0]	[0.7–1.4]
≥65	0.5	0.4	0.6	0.4	0.6	0.4	0.9	1.3	1.1	0.9	0.8
	[0.3–0.7]	[0.2–0.6]	[0.4–0.8]	[0.3–0.6]	[0.4–0.8]	[0.2–0.6]	[0.6–1.1]	[1.0–1.6]	[0.8–1.4]	[0.6–1.2]	[0.6–1.0]
