# Supplementary material for: The changing face of nicotine use in England: Age‐specific annual trends, 2014 to 2024
Source: Addiction. 2025 Dec 7;121(3):549–63. doi: 10.1111/add.70243 (PMC12887924; doi:10.1111/add.70243)
Supplement: Supplementary file 5 — Data S5. Supplementary Information. [file ADD-121-549-s002.pdf]

**Supplementary File 5: Smoking history among adults who vape**

**Table S19.** Proportion who have never regularly smoked among adults who vape, by age group and year

| | Never regularly smoked, % [95% confidence interval] | | | | | | | | | | |
|---|---|---|---|---|---|---|---|---|---|---|---|
| | **2014** | **2015** | **2016** | **2017** | **2018** | **2019** | **2020** | **2021** | **2022** | **2023** | **2024** |
| 18-24 | 4.3 | 1.9 | 2.6 | 5.5 | 7.2 | 9.2 | 15.1 | 14.3 | 19.1 | 28.6 | 34.3 |
| | [0.9–7.6] | [0.0–4.2] | [0.3–5.0] | [1.4–9.5] | [2.3–12.1] | [4.0–14.4] | [9.4–20.8] | [9.3–19.3] | [15.0–23.3] | [24.3–33.0] | [29.8–38.8] |
| 25-34 | 1.5 | 3.9 | 4.2 | 8.4 | 3.2 | 9.1 | 5.4 | 4.5 | 10.3 | 11.1 | 13.3 |
| | [0.0–3.2] | [0.9–6.9] | [1.5–6.9] | [4.4–12.4] | [0.6–5.9] | [5.0–13.2] | [2.4–8.4] | [2.1–6.9] | [7.3–13.3] | [8.2–14.0] | [10.3–16.2] |
| 35-44 | 4.5 | 1.0 | 5.4 | 7.3 | 7.2 | 5.7 | 2.7 | 4.8 | 5.9 | 8.4 | 9.6 |
| | [1.1–8.0] | [0.0–2.2] | [2.2–8.7] | [3.3–11.4] | [3.4–10.9] | [2.3–9.0] | [0.5–4.9] | [1.9–7.8] | [3.1–8.8] | [5.4–11.5] | [6.4–12.8] |
| 45-54 | 2.9 | 5.8 | 3.9 | 5.6 | 6.2 | 7.0 | 5.0 | 3.7 | 7.0 | 5.6 | 6.3 |
| | [0.5–5.2] | [2.0–9.6] | [1.2–6.6] | [2.4–8.7] | [2.8–9.6] | [3.5–10.4] | [2.1–8.0] | [1.2–6.3] | [3.7–10.4] | [2.8–8.4] | [3.3–9.3] |
| 55-64 | 1.6 | 1.6 | 4.8 | 3.3 | 5.1 | 4.8 | 6.5 | 3.1 | 1.6 | 5.1 | 4.5 |
| | [0.0–3.5] | [0.0–3.3] | [1.5–8.2] | [0.7–5.9] | [1.6–8.7] | [1.7–7.9] | [2.5–10.4] | [0.4–5.9] | [0.0–3.4] | [2.2–7.9] | [1.7–7.3] |
| ≥65 | 1.9 | 6.2 | 4.5 | 3.8 | 5.4 | 7.9 | 8.3 | 4.6 | 3.8 | 2.1 | 4.2 |
| | [0.0–4.1] | [0.0–12.4] | [0.9–8.2] | [0.2–7.5] | [1.3–9.5] | [2.8–13.1] | [3.7–13.0] | [0.5–8.7] | [0.0–7.7] | [0.0–4.3] | [0.5–7.9] |

**Table S20.** Proportion who quit smoking ≥1 year ago among adults who vape, by age group and year

| | Quit smoking ≥1 year ago, % [95% confidence interval] | | | | | | | | | | |
|---|---|---|---|---|---|---|---|---|---|---|---|
| | **2014** | **2015** | **2016** | **2017** | **2018** | **2019** | **2020** | **2021** | **2022** | **2023** | **2024** |
| 18-24 | 5.1 | 5.5 | 7.0 | 9.1 | 7.2 | 13.8 | 12.9 | 14.6 | 13.1 | 12.4 | 19.3 |
| | [0.9–9.3] | [1.5–9.5] | [3.1–10.9] | [4.3–13.9] | [2.9–11.5] | [7.1–20.5] | [7.3–18.5] | [9.6–19.7] | [9.5–16.7] | [9.0–15.9] | [15.4–23.1] |
| 25-34 | 10.7 | 14.9 | 15.8 | 23.7 | 25.5 | 25.7 | 31.5 | 26.4 | 28.9 | 31.0 | 35.5 |
| | [6.0–15.3] | [9.6–20.1] | [10.9–20.7] | [17.8–29.6] | [19.6–31.4] | [19.2–32.1] | [25.3–37.6] | [21.1–31.8] | [24.3–33.4] | [26.7–35.3] | [31.3–39.7] |
| 35-44 | 9.6 | 18.8 | 24.4 | 28.6 | 27.4 | 36.2 | 47.4 | 36.8 | 42.9 | 37.7 | 44.8 |
| | [5.1–14.0] | [12.9–24.7] | [17.7–31.2] | [21.9–35.2] | [20.6–34.1] | [28.3–44.0] | [39.9–54.9] | [29.7–43.9] | [36.6–49.1] | [32.6–42.9] | [39.6–49.9] |
| 45-54 | 10.9 | 14.7 | 26.7 | 29.9 | 35.2 | 35.7 | 38.6 | 42.5 | 38.3 | 48.9 | 45.9 |
| | [6.2–15.5] | [9.7–19.6] | [20.5–32.9] | [23.4–36.4] | [28.3–42.2] | [28.4–43.0] | [31.5–45.8] | [35.6–49.4] | [31.9–44.7] | [43.2–54.7] | [39.8–52.0] |
| 55-64 | 15.7 | 13.2 | 25.2 | 33.8 | 30.1 | 39.9 | 45.0 | 44.6 | 41.9 | 40.6 | 54.2 |
| | [8.5–23.0] | [7.5–18.9] | [18.1–32.3] | [26.2–41.3] | [22.6–37.5] | [32.1–47.6] | [36.9–53.1] | [37.2–52.1] | [33.8–50.0] | [34.4–46.8] | [47.5–60.8] |
| ≥65 | 13.9 | 19.1 | 24.2 | 32.8 | 41.7 | 37.1 | 40.3 | 46.3 | 48.2 | 52.1 | 55.2 |
| | [7.0–20.8] | [11.0–27.2] | [16.3–32.1] | [23.9–41.8] | [32.8–50.6] | [27.4–46.8] | [30.7–49.9] | [37.2–55.4] | [37.9–58.4] | [43.5–60.8] | [47.1–63.3] |

**Supplementary File 5: Smoking history among adults who vape**

**Table S21.** Proportion who quit smoking <1 year ago among adults who vape, by age group and year

| | Quit smoking <1 year ago, % [95% confidence interval] | | | | | | | | | | |
|---|---|---|---|---|---|---|---|---|---|---|---|
| | **2014** | **2015** | **2016** | **2017** | **2018** | **2019** | **2020** | **2021** | **2022** | **2023** | **2024** |
| 18-24 | 6.2 | 10.2 | 9.9 | 10.5 | 12.6 | 5.5 | 9.9 | 11.9 | 13.2 | 9.8 | 8.5 |
| | [2.1–10.3] | [4.8–15.7] | [5.4–14.4] | [5.1–15.9] | [6.1–19.1] | [1.7–9.3] | [5.0–14.8] | [7.4–16.4] | [9.6–16.9] | [6.8–12.9] | [5.8–11.3] |
| 25-34 | 10.6 | 10.2 | 14.4 | 10.8 | 9.2 | 6.8 | 10.6 | 12.5 | 10.5 | 11.4 | 11.7 |
| | [5.8–15.4] | [5.7–14.7] | [8.8–20.0] | [6.1–15.6] | [5.4–13.0] | [2.6–11.0] | [6.8–14.4] | [8.5–16.4] | [7.6–13.3] | [8.6–14.3] | [8.8–14.7] |
| 35-44 | 18.9 | 13.1 | 11.4 | 7.0 | 8.6 | 4.7 | 8.9 | 9.4 | 10.8 | 11.3 | 10.0 |
| | [12.7–25.1] | [7.8–18.3] | [6.8–16.0] | [3.2–10.7] | [4.2–13.0] | [1.0–8.5] | [4.8–13.1] | [5.0–13.8] | [6.9–14.8] | [7.9–14.8] | [6.9–13.0] |
| 45-54 | 10.9 | 13.6 | 10.4 | 11.1 | 6.4 | 7.2 | 7.3 | 8.0 | 7.4 | 6.0 | 6.4 |
| | [6.0–15.9] | [8.5–18.8] | [6.1–14.7] | [6.5–15.7] | [3.0–9.8] | [3.3–11.1] | [3.3–11.2] | [4.1–11.8] | [4.0–10.9] | [3.5–8.6] | [3.6–9.2] |
| 55-64 | 9.0 | 8.7 | 9.6 | 8.2 | 4.9 | 5.5 | 1.7 | 7.8 | 6.5 | 8.1 | 6.8 |
| | [3.8–14.3] | [4.0–13.4] | [4.7–14.4] | [4.0–12.4] | [1.5–8.4] | [1.5–9.6] | [0.0–3.4] | [3.8–11.8] | [2.3–10.6] | [4.4–11.9] | [3.6–10.1] |
| ≥65 | 10.5 | 8.1 | 11.3 | 8.1 | 1.2 | 3.9 | 3.6 | 4.0 | 10.6 | 6.6 | 5.1 |
| | [4.9–16.2] | [1.8–14.3] | [5.2–17.5] | [3.1–13.1] | [0.0–2.8] | [0.4–7.3] | [0.0–7.1] | [0.0–8.0] | [4.7–16.5] | [2.1–11.1] | [1.4–8.8] |

**Table S22.** Proportion who currently smoke among adults who vape, by age group and year

| | Currently smoke, % [95% confidence interval] | | | | | | | | | | |
|---|---|---|---|---|---|---|---|---|---|---|---|
| | **2014** | **2015** | **2016** | **2017** | **2018** | **2019** | **2020** | **2021** | **2022** | **2023** | **2024** |
| 18-24 | 84.4 | 82.4 | 80.5 | 74.9 | 72.9 | 71.6 | 62.1 | 59.2 | 54.5 | 49.1 | 37.9 |
| | [78.1–90.8] | [75.7–89.2] | [74.5–86.5] | [67.3–82.5] | [64.7–81.2] | [63.4–79.7] | [54.0–70.2] | [52.2–66.1] | [49.3–59.8] | [44.3–54.0] | [33.3–42.5] |
| 25-34 | 77.3 | 71.1 | 65.5 | 57.1 | 62.1 | 58.4 | 52.6 | 56.6 | 50.4 | 46.4 | 39.4 |
| | [71.0–83.6] | [64.4–77.8] | [58.6–72.4] | [50.0–64.1] | [55.5–68.6] | [51.2–65.6] | [45.9–59.2] | [50.6–62.6] | [45.4–55.4] | [41.7–51.1] | [35.0–43.9] |
| 35-44 | 67.0 | 67.2 | 58.7 | 57.2 | 56.9 | 53.4 | 41.0 | 48.9 | 40.4 | 42.5 | 35.7 |
| | [59.6–74.4] | [60.0–74.3] | [51.3–66.1] | [49.9–64.4] | [49.4–64.3] | [45.4–61.5] | [33.5–48.4] | [41.6–56.3] | [34.2–46.5] | [37.2–47.7] | [30.6–40.7] |
| 45-54 | 75.3 | 65.9 | 59.0 | 53.5 | 52.2 | 50.1 | 49.1 | 45.8 | 47.2 | 39.4 | 41.4 |
| | [68.7–81.9] | [59.0–72.9] | [52.1–65.8] | [46.5–60.6] | [45.1–59.3] | [42.4–57.8] | [41.6–56.6] | [38.8–52.8] | [40.6–53.9] | [33.8–45.0] | [35.4–47.5] |
| 55-64 | 73.6 | 76.5 | 60.4 | 54.7 | 59.9 | 49.8 | 46.8 | 44.4 | 50.0 | 46.2 | 34.5 |
| | [65.1–82.1] | [69.4–83.5] | [52.5–68.3] | [46.8–62.6] | [51.9–67.8] | [42.0–57.7] | [38.7–54.9] | [36.9–52.0] | [41.7–58.4] | [39.8–52.5] | [28.1–40.9] |
| ≥65 | 73.7 | 66.6 | 60.0 | 55.3 | 51.8 | 51.1 | 47.8 | 45.1 | 37.4 | 39.1 | 35.5 |
| | [65.1–82.2] | [56.2–77.0] | [50.8–69.2] | [45.8–64.7] | [42.7–60.8] | [41.1–61.0] | [38.1–57.5] | [36.0–54.2] | [27.6–47.2] | [30.6–47.6] | [27.7–43.4] |
